# High-Performance Triboelectric Nanogenerators Based on Triazine-Based Covalent Organic Framework/PVDF-HFP Composite Nanofibrous Membranes for Wearable Electronics

**DOI:** 10.1021/acspolymersau.6c00106

**Published:** 2026-07-27

**Authors:** Ying-Hao Pan, Hsin-Jung Kan, Pei-Cih Hu, Hongta Yang, Rong-Ho Lee

**Affiliations:** † Department of Chemical Engineering, 34916 National Chung Hsing University, Taichung 402, Taiwan; ‡ Department of Chemical Engineering and Materials Science, Yuan Ze University, Taoyuan 320, Taiwan

**Keywords:** covalent organic framework, TPU, PVDF-HFP, electrospinning, triboelectric nanogenerator

## Abstract

In this study, triazine-based covalent organic framework (COF)/poly­(vinylidene fluoride-*co*-hexafluoropropylene) (COF/PVDF-HFP) nanofibrous membranes were fabricated via electrospinning and utilized as the negative triboelectric layer in a triboelectric nanogenerator (TENG). The large electronegativity difference between PVDF-HFP and the COF gives rise to an interfacial p–n heterostructure. The polarity disparity at the interface intensifies charge transfer during frictional interactions, leading to increased charge generation. The TPTP-COF- and TPBT-COF-based TENGs generated higher open-circuit voltages (*V*_OC_) and short-circuit current densities (I_SC_) values compared to the TPDA-COF- and TPBTz-COF-based devices, owing to their larger specific surface areas, higher dielectric constants, and more efficient surface charge generation. The optimized TPBT-COF/PVDF-HFP membrane-based TPBT-1.5 TENG achieved maximum *V*_OC_, I_SC_, and transferred charge of 338.2 V, 74.0 μA, and 127.1 nC, respectively. A maximum output power density of 16.76 W m^–2^ was achieved at an external load resistance of 50 MΩ. Under operating conditions of 60 N applied force at 5 Hz, the COF/PVDF-HFP-based TENG successfully powered LEDs while maintaining stable Voc performance over 20,000 contact–separation cycles. Furthermore, four TPBT-1.5 TENGs connected in series were attached to a custom-made insole and placed inside a shoe. A researcher wore the shoe and performed jogging to generate electrical energy, which was stored in a connected capacitor and subsequently used to power an LED. The developed COF/PVDF-HFP TENG can function as a walk-induced energy-harvesting device, serving as a practical power source for wearable electronic devices.

## Graphical abstract

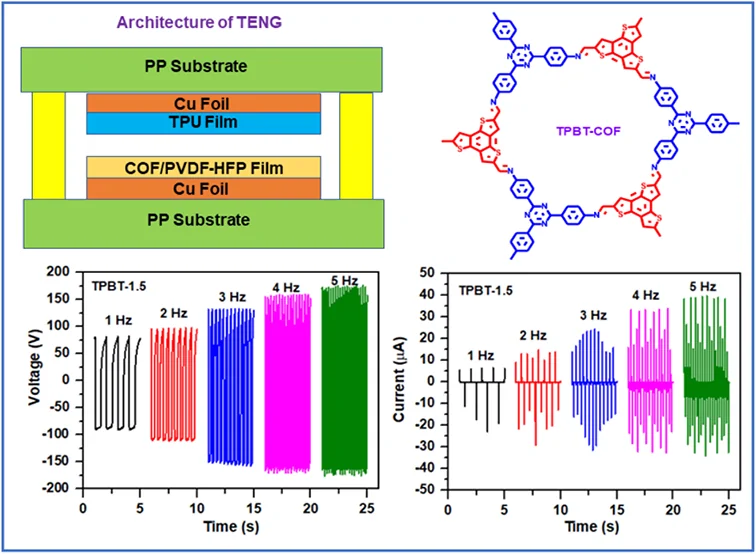

## 1 Introduction

With the increasingly widespread application of intelligent electronics, triboelectric nanogenerators (TENGs) have recently attracted considerable attention as promising sustainable micropower sources. In particular, flexible and stretchable TENGs have drawn significant interest owing to their lightweight nature, low fabrication cost, and high energy-conversion efficiency.^1–3^ A TENG operates by coupling triboelectrification with electrostatic induction to convert mechanical stimuli into electrical energy. In a contact–separation mode TENG, the two dielectric layers act as oppositely charged surfaces—one tribopositive and the other tribonegative—due to differences in electron affinity arising from variations in their molecular and material structures. Consequently, one surface donates electrons while the other accepts them, thereby generating and sustaining triboelectric surface charges throughout repeated contact–separation cycles.^2,3^ The positive triboelectric layers are polymers containing functional groups with lone electron pairs, such as polyurethane, poly­(vinyl alcohol) (PVA), polyamide, and poly­(methyl methacrylate).^3^ Conversely, negative triboelectric layers typically contain strongly electronegative functional groups, including poly­(vinylidene fluoride-*co*-hexafluoropropylene) (PVDF-HFP), poly­(vinylidene fluoride) (PVDF), polytetrafluoroethylene (PTFE), and polyacrylonitrile.^3^ To enhance the electronegativity and charge-storage capability of negative triboelectric layers, various nanomaterials—such as CNTs, rGO, graphene, graphene quantum dots, MXenes, metals, transition-metal oxides, metal sulfides, metal nitrides, and metal hydroxides—have been incorporated into these layers.^4–11^ The introduction of polar nanomaterials induces interfacial polarization, which increases the dielectric constant and consequently improves the electrical output performance of TENGs.

Covalent organic frameworks (COFs) exhibit high crystallinity, large specific surface area, excellent structural stability, tunable architectures, and versatile chemical functionalities, which have led to their extensive study for applications in energy conversion and storage, drug delivery, adsorption of various pollutants, gas separation, catalysis, and sensing.^12–22^ The structurally predesignable, synthetically controllable, and functionally tailorable nature of COFs makes them an ideal platform for exploring the fundamental physical and chemical mechanisms in a variety of device systems. A highly fluorinated (COF) Tp-TFAB is successfully synthesized and utilized as positive triboelectric materials for high-performance TENGs.^23^ Compared with the nonfluorinated Tp-TAPB COF, both the pristine Tp-TFAB COF and corresponding hybrid films with PVA based TENGs demonstrate much higher triboelectric performance. Detailed investigations revealed that the fluorinated Tp-TFAB COF has enhanced electron donating ability, which significantly boosts the triboelectric output of TENGs. A COF functionalized with electron-withdrawing bromine groups on its skeleton has been synthesized for TENG applications.^24^ The bromine-functionalized COF exhibits more regularly aligned π-columns and ordered arrays along the skeleton compared to the unmodified COF, significantly enhancing charge transport. Consequently, the bromine-functionalized COF demonstrates higher electrical output performance than the bare COF. A series of COFs with similar skeletons but different functional groups, ranging from electron-donating to electron-withdrawing, were incorporated into PVDF films for TENG applications.^25^ The surface potentials and electron affinities of the COFs can be tuned by introducing these functional groups. Among them, the TENG containing the fluorinated COF exhibited superior triboelectric output compared to other reported materials. Two COFs with similar topology and skeletons but different charge states were designed to investigate the effect of cationic groups on TENG performance.^26^ The cationic functional centers, distributed alternately along both sides of the pore channels, induce unconventional electrostatic behavior via ionic interfaces. When integrated into a dipole-based TENG, the cationic COF achieves approximately twice the output of its neutral counterpart. A metal-functionalized COF (Cu-COF) was designed and synthesized via a postmetalation approach, in which transition metal ions were introduced into the chelating sites of a CF_3_-substituted covalent organic framework (CF_3_–COF) for TENG applications.^27^ The resulting Cu-COF exhibited enhanced triboelectric nanogenerator performance, which can be attributed to its high charge density, improved conductivity, and narrow band gap. Most studies directly disperse and adsorb COFs onto Cu or Ni foils to serve as the positive triboelectric layer in TENGs.^14,23,24,26–28^ Moreover, prior research has predominantly focused on introducing functional groups into COFs to enhance their electrostatic generation capability, while comparatively few studies have explored how COFs with different conjugated structures and donor–acceptor characteristics influence the electrostatic performance of triboelectric generators.

In this study, thermoplastic polyurethane (TPU) nanofibrous membranes were fabricated via electrospinning as the positive friction layer, while COF (TPTP-COF, TPBT-COF, TPDA-COF, and TPBTz-COF)/PVDF-HFP composite nanofibrous membranes were prepared as the negative friction layer. These components were subsequently assembled into a TENG. Incorporating the conjugated COF with electron-rich units into the PVDF-HFP nanofibrous membrane would induce interfacial polarization under an applied electric field, thereby increasing the dielectric constant and promoting surface charge generation during contact–separation cycles. Moreover, compared with the triazine-based TPTP-COF, the dielectric properties and surface potential of TPBT-COF are further modulated by the donor–acceptor structure formed through the incorporation of triazine and benzotrithiophene units. Incorporating the electron-withdrawing anthraquinone unit into the triazine-based COF increases the electronegativity of the TPDA-COF/PVDF-HFP composite film, which is favorable for charge transfer from the positive triboelectric layer. In contrast, introducing the electron-donating benzobisthiazole moiety into the triazine-based COF enhances intraframework charge transfer and conjugation within the TPBTz-COF, thereby improving the charge-generation capability of the TPBTz-COF/PVDF-HFP composite film. Overall, these four triazine-based COFs, functionalized with different electron-donating and electron-withdrawing units on the backbone, were incorporated into the PVDF-HFP membrane to systematically investigate how chemical structure influences the triboelectric output performance. SEM and TEM analyses showed that the incorporation of COFs had no significant effect on the fiber diameter, while TEM offered higher-resolution insights into the morphology of the COF/PVDF-HFP nanofibers. XRD and FTIR analyses were used to investigate the crystallization characteristics of the membranes. The COF/PVDF-HFP-based TENGs exhibited superior triboelectric output compared to those based solely on PVDF-HFP membranes. The triboelectric output of PVDF-HFP-based TENGs was greatly enhanced by COF incorporation. Moreover, the TPTP-COF and TPBT-COF-based TENGs exhibited higher triboelectric performance compared to the TPDA-COF and TPBTz-COF-based TENGs. In addition to the chemical structure, the specific surface area of the COF also plays a crucial role in determining the dielectric properties, surface potential, and overall triboelectric performance of the COF/PVDF-HFP-based TENG. The optimized TPBT-COF/PVDF-HFP membrane based TPBT-1.5 TENG achieved maximum open-circuit voltages (*V*_OC_), short-circuit current densities (I_SC_), and transferred charge of 338.2 V, 74.0 μA, and 127.1 nC, respectively. A maximum output power density of 16.76 W m^–2^ was achieved at an external load resistance of 50 MΩ. Under operating conditions of 60 N applied force at 5 Hz, the COF/PVDF-HFP-based TENG successfully powered LEDs, while maintaining stable *V*_OC_ performance over 20,000 contact–separation cycles. Furthermore, four TPBT-1.5 TENGs connected in series were attached to a custom-made insole and placed inside a shoe. A researcher wore the shoe and performed jogging to generate electrical energy, which was then stored in a connected capacitor and subsequently used to power an LED. This study confirms that the developed COF/PVDF-HFP TENG, when installed in an insole, can be applied as a walk-induced energy-harvesting device for athletes or hikers, serving as a power source for wearable electronic devices.

## 2 Experimental Details

### 2.1 Chemicals and COF Materials

N,N-Dimethylformamide (DMF) and tetrahydrofuran (THF) were purchased from Aldrich. Thermoplastic polyurethane (TPU; Mn = 90,000 g·mol^–1^, Mw = 200,000 g·mol^–1^) pellet was purchased from Covestro Ltd., and poly­(vinylidene fluoride-*co*-hexafluoropropylene) (PVDF-HFP; Mn = 130,000 g·mol^–1^, Mw = 40,0000 g·mol^–1^) pellet was purchased from Sigma-Aldrich. TPTP-COF, TPBT-COF, TPDA-COF, and TPBTz-COF were synthesized following previously reported methods.^12,16^ The chemical structures of the four COFs are illustrated in Figure 1.

**Figure 1:**
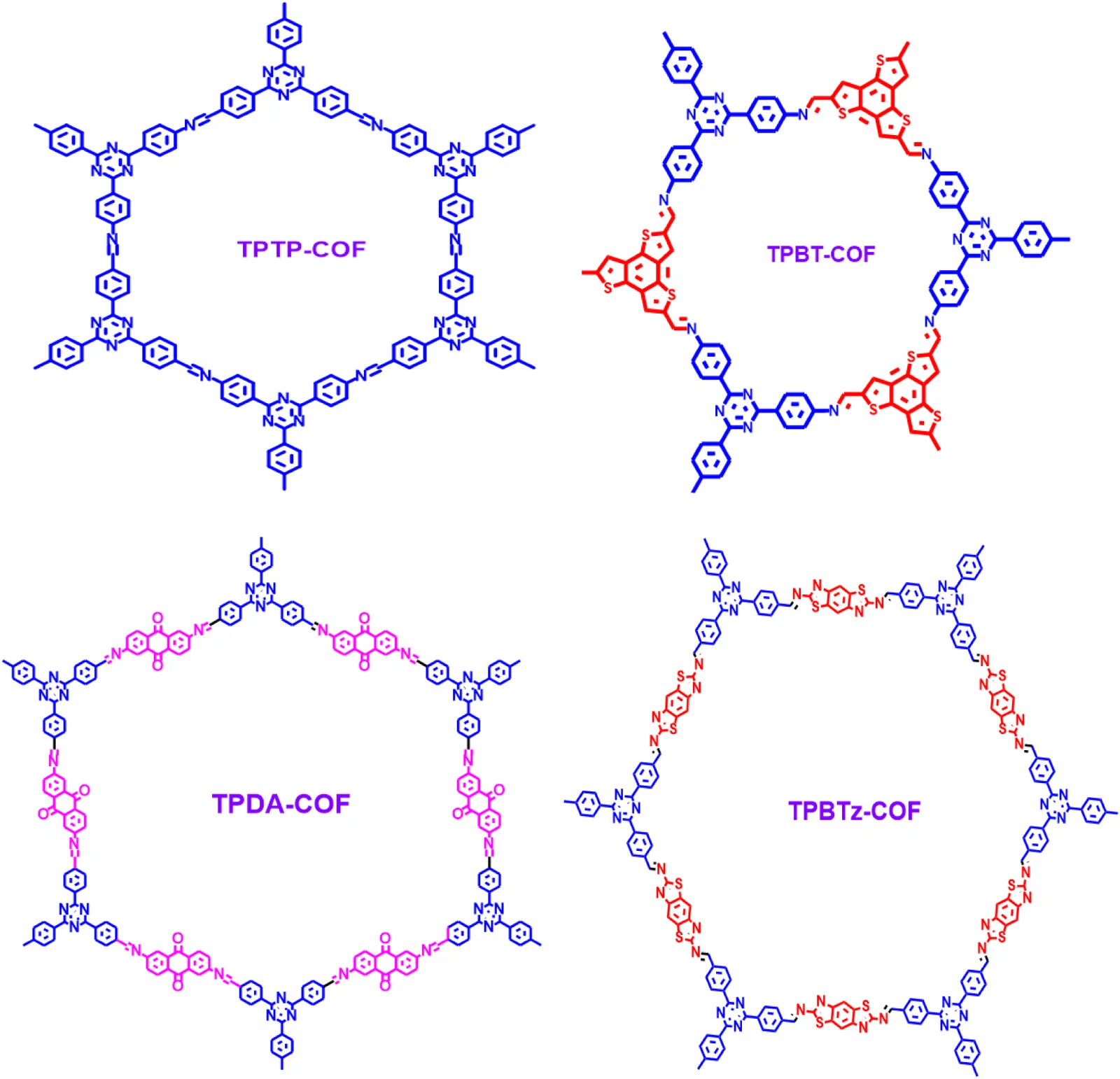
Chemical structures of the TPTP-COF, TPBT-COF, TPDA-COF, and TPBTz-COF.

### 2.2 Preparation of TPU and COF/PVDF-HFP-Based Electro-Spun Nanofibrous Membranes

The TPU nanofibrous membrane was fabricated using vertical electrospinning equipment (FES-COS, Taiwan). TPU pellets were dissolved in a DMF/THF (1:1, v/v) mixed solvent (12 wt %) to prepare the electrospinning solution, which was loaded into a plastic syringe equipped with a 19-gauge stainless steel needle. The solution was delivered at a constant flow rate of 1.4 mL h^–1^ using a syringe pump (NE-300 Just Infusion, USA). The electrospinning nozzle was moved laterally over a distance of 18 cm at a speed of 4.5 cm s^–1^. A voltage of 8.5 kV was applied, with a tip-to-collector distance of 14 cm, and fibers were collected on a baking paper wrapped stationary metal plate (25 × 25 cm^2^; baking paper thickness ∼0.5 mm) for 4 h. The electrospinning process was carried out at 27.5 ± 2.5 °C and 45 ± 5% relative humidity (RH). The obtained membranes were dried in an oven at 60 °C for 6 h.

For COF/PVDF-HFP nanofibrous membranes, COF powder (0.5, 1.0, 1.5, and 2.0 wt % relative to PVDF-HFP) was dispersed in a 19 wt % PVDF-HFP solution prepared in DMF (12 mL). The electrospinning solution was loaded into a syringe with a 21-gauge stainless steel needle and fed at 1.4 mL h^–1^. A voltage of 14 kV was applied, with a tip-to-collector distance of 18 cm. Fibers were collected for 4 h on a baking paper wrapped rotating drum (1500 rpm) to obtain aligned nanofibers. The process was conducted under the same temperature and RH conditions as above, and membranes were dried at 60 °C for 6 h.

### 2.3 Characterization of TPU and COF/PVDF-HFP Nanofibrous Membranes

Fourier transform infrared (FTIR) spectra were recorded using a HORIBA FT-720 spectrometer. The dynamic light scattering (DLS) method (Litesizer DLS 100) was used to measure the particle size distribution of the COFs (1 mg) in 10 mL of DMF solvent. The DLS measurement samples were prepared by subjecting the COF solution to ultrasonic treatment at room temperature for a duration of 2 h. The UV–Vis absorption spectra of the COF/PVDF-HFP nanofibrous membranes were recorded using a Hitachi U3010 UV–Vis spectrophotometer. Thermogravimetric analysis (TGA) was performed using a PerkinElmer Pyris system to evaluate the thermal stability of the COF/PVDF-HFP nanofibrous membranes. The measurements were conducted under a nitrogen atmosphere at a heating rate of 20 °C min^–1^, and the thermal decomposition temperatures were determined from the resulting TGA curves. Ultraviolet photoelectron spectroscopy (UPS) measurements were performed using a PHI 5000 VersaProbe III (ULVAC-PHI Inc.) equipped with a He I (21.2 eV) excitation source to determine the valence band maximum (VBM), conduction band minimum (CBM), and work function (WF) of the films. Surface morphologies were examined by cold field emission scanning electron microscopy (FESEM; Hitachi-4800) operated at 1.5–2.0 kV. High-resolution transmission electron microscopy (HRTEM, JEOL JEM-1400Flash, Japan) operated at 200 kV was used to observe the morphology of COF/PVDF-HFP nanofibrous membranes, which were collected on Cu grids. The crystalline structures of the membranes were characterized by X-ray diffraction (XRD, Shimadzu SD-D1) using a Cu target at 35 kV and 30 mA. The water contact angles (CAs) of COF/PVDF-HFP nanofibrous membranes were measured using a Kyowa DropMaster optical contact angle meter. The dielectric constants of the COF/PVDF-HFP composite films were measured using an impedance analyzer (Microtest 6632) over a frequency range of 10 Hz to 50 MHz. The surface roughness (Ra), contact potential difference (CPD), and work function (Φ) of the nanofibrous films were measured using KPFM (Bruker, Dimension Icon).

### 2.4 Fabrication of the TPU and COF/PVDF-HFP Nanofibrous Membrane-Based TENGs

The architecture of the TPU- and COF/PVDF-HFP-based nanofibrous membrane TENGs is shown in Figure 2. To fabricate the TENG, TPU and COF/PVDF-HFP nanofibrous membranes (3.0 × 2.0 cm^2^, average thickness ∼0.3 mm) were cut and affixed to commercially available copper (Cu) foil tapes, which served as electrodes for the positive and negative friction layers. Each triboelectric-layer-adhered Cu foil was then attached to a polypropylene (PP) substrate using double-sided adhesive tape. The two PP substrates bearing the positive and negative triboelectric layers were assembled into a TENG device using four PU foam spacers, with a separation distance of approximately 10 mm between them.

**Figure 2:**
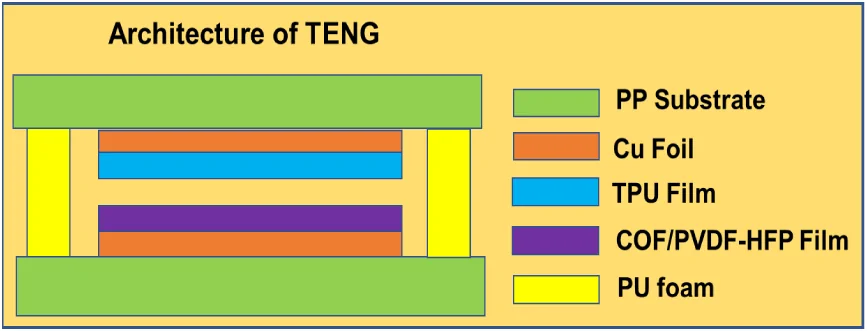
Architecture of the TPU and COF/PVDF-HFP nanofibrous membranes based TENG.

### 2.5 Triboelectric Characterization of the TENGs

The working principle of the vertical contact–separation mode TENG is illustrated in Figure 3.^1^ Two dielectric friction layers with electrodes are placed face-to-face. Upon contact, opposite charges are generated on the two layers via the triboelectric effect. When the layers separate, an electrostatic field forms between them, creating a potential difference that drives electrons through an external circuit. This repetitive contact–separation process produces alternating current (AC) electricity.^1^ The *V*_OC_ was measured using a Keithley 6514 electrometer, while the I_SC_ was recorded via an oscilloscope connected to a Stanford Research Systems SR570 current preamplifier. A LinMot linear motor system supplied a stable external force to induce reciprocating contact between the TPU- and PVDF-HFP-based electrodes. Dynamic mechanical pressure was applied using a Montrol motion control system (MSP 50072) under various forces (10–70 N) and frequencies (1–10 Hz). To simulate actual operating conditions, the contact–separation distance between the triboelectric layers was fixed at 10 mm. Transferred charge was measured with the Keithley 6514 electrometer. All measurements were performed under ambient conditions.

**Figure 3:**
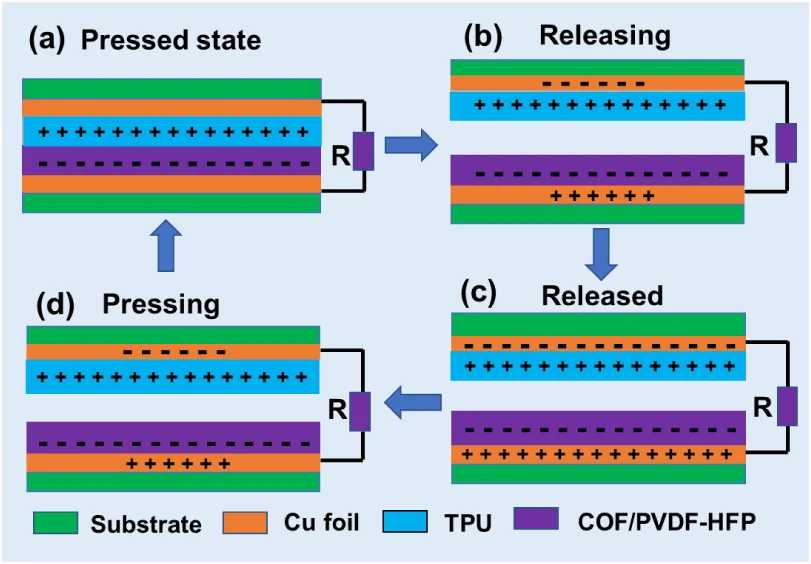
Schematic illustration of the working mechanism of the TENG device.

## 3 Results and Discussion

### 3.1 Characterization of the COF Nanomaterials

As shown in Figure S1, all COF materials exhibited nanoscale dimensions after ultrasonic treatment. TPTP-COF and TPBT-COF displayed spherical aggregates with an average particle size of 273 and 332 nm. TPDA-COF and TPBTz-COF exhibited rod-like morphologies with average particle sizes of 264 and 158 nm, respectively. Moreover, the particle size distribution of TPBTz-COF was significantly narrower than those of TPTP-COF, TPBT-COF, and TPDA-COF. In addition, the specific surface areas of the COFs were determined using the Brunauer–Emmett–Teller (BET) method, yielding values of 1523.5, 1163.5, 28.4, and 20.3 m^2^ g^–1^ for TPTP-COF, TPBT-COF, TPDA-COF, and TPBTz-COF, respectively.^12,16^ The combination of smaller particle size and larger specific surface area facilitates the dispersion of COFs within the electrospun nanofibrous membranes, thereby enhancing the triboelectric performance of the nanogenerators.

The UV–vis absorption spectra of the samples are shown in Figure S2a. The absorption edges of TPTP-COF, TPBT-COF, TPDA-COF, and TPBTz-COF extend to 556, 709, 554, and 670, respectively.^12^ TPBT-COF and TPBTz-COF exhibit broader absorption bands than TPTP-COF and TPDA-COF, with markedly enhanced absorption in the 400–800 nm region, demonstrating superior visible-light harvesting capability. The donor–acceptor framework promotes π-electron delocalization and intramolecular charge transfer, thereby enhancing the conjugation effect in TPBT-COF and TPBTz-COF. Based on the Tauc plots shown in Figure S2b, the optical band gaps of TPTP-COF, TPBT-COF, TPDA-COF, and TPBTz-COF were estimated to be 2.23, 1.75, 2.24, and 1.85 eV, respectively. Compared with the triazine-based TPTP-COF, the incorporation of electron-donating building units into the core and bridge structures effectively narrows the band gaps of TPBT-COF and TPBTz-COF, respectively. In contrast, the introduction of an electron-withdrawing bridge unit in TPDA-COF leads to a slightly wider band gap. Notably, TPBT-COF exhibits the narrowest band gap (1.75 eV), corresponding to an absorption edge of approximately 708 nm. The energy band structures of the TPTP-COF, TPBT-COF, TPDA-COF, and TPBTz-COF were further investigated using UPS spectroscopy. The WF, VBM, and CBM were calculated using the following equations: WF = 21.22 – E_cutoff_ and VBM = WF + E_onset_, where E_cutoff_ and E_onset_ are obtained from FiguresS2c and S2d, respectively.^29^ The UPS-derived energy level values are summarized in Table S1. The VBM values referenced to the vacuum level are −5.82, −5.72, −5.77, and −5.80 eV for TPTP-COF, TPBT-COF, TPDA-COF, and TPBTz-COF, respectively. The corresponding CBM values, calculated using the relation CBM = VBM −Eg are −3.59, −3.97, −3.53, and −3.95 eV, respectively. The narrower energy bandgaps of TPBT-COF and TPBTz-COF facilitate charge transfer within the COF/PVDF-HFP nanomembranes, thereby enhancing charge transport efficiency.

### 3.2 Thermal Properties and Morphology of the COF/PVDF-HFP Nanofibrous Membranes

The TGA curves of the COF/PVDF-HFP nanofibrous membranes are presented in Figure S3. All of the COF/PVDF-HFP nanofibrous membranes exhibit thermal decomposition temperatures above 450 °C, indicating excellent thermal stability. Moreover, the char yields of the COF/PVDF-HFP nanofibrous membranes are slightly higher than that of the pristine PVDF-HFP nanofibrous membrane, suggesting that the incorporation of COFs slightly enhances the thermal stability of the nanofibrous membranes.

The morphology and fiber diameter distributions of TPU and aligned PVDF-HFP nanofibrous membranes are shown in Figure S4. TPU nanofibers exhibited significantly larger diameters (1200–2800 nm) compared to aligned PVDF-HFP (100–450 nm) nanofibers. SEM images revealed well-defined unidirectional alignment for aligned PVDF-HFP nanofibers (Figure S4b). Similarly, aligned COF/PVDF-HFP nanofibers (TPTP-COF/PVDF-HFP, TPBT-COF/PVDF-HFP, TPDA-COF/PVDF-HFP, TPBTz-COF/PVDF-HFP) showed good unidirectional alignment. The fiber diameters were distributed in the range of 50–550 nm, remaining nearly unchanged as the COF content increased (Figure 4 and Figures S5–S12). As shown in Figure S13, the fiber diameter distributions of the TPTP-1.5 and TPBT-1.5 membranes were slightly larger than that of the TPDA-1.0 membrane. Moreover, the TPBTz-1.5 membrane exhibited the largest fiber diameters among all the samples. In addition, SEM, TEM, and EDX analyses of COF/PVDF nanofibrous membranes confirmed the uniform distribution of COF particles throughout the PVDF-HFP nanofibrous matrix (Figure 5, Figure S14, and Figures S15–17). The detection of N, S, and O elements in the EDX images of the COF/PVDF-HFP membranes confirms the uniform dispersion of COF nanoparticles throughout the PVDF-HFP electrospun fibers.

**Figure 4:**
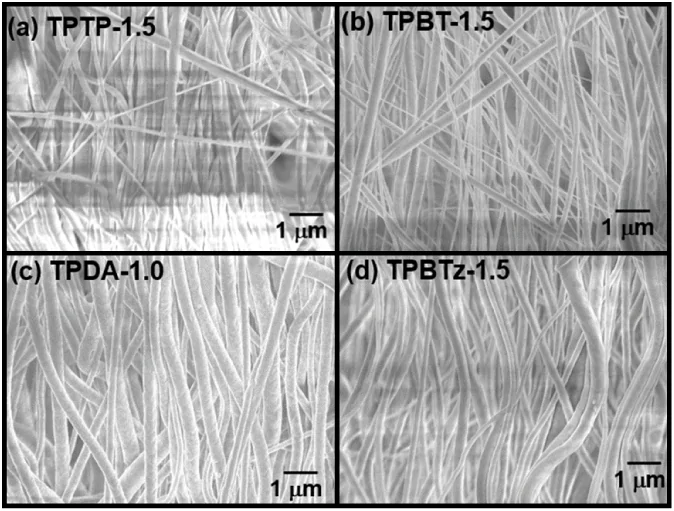
SEM images of the (a) TPTP-1.5, (b) TPBT-1.5, (c) TPDA-1.0, and (d) TPBTz-1.5 nanofibrous membranes.

**Figure 5:**
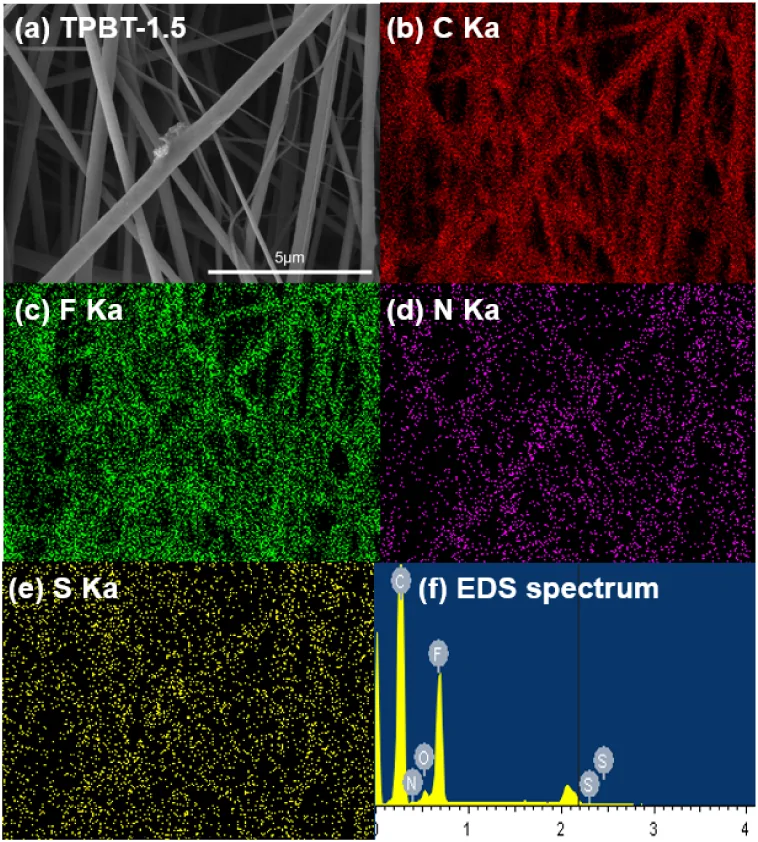
(a) SEM image, (b–e) EDX images of (b) C, (c) F, (d) N, (e) S, and (f) EDS spectrum in the TPBTz-1.5 nanofibrous membranes.

### 3.3 Crystal Structure of COF/PVDF-HFP Nanofibrous Membranes

Figure 6 shows the FTIR spectra of PVDF-HFP, and COF/PVDF-HFP (TPTP-COF/PVDF-HFP, TPBT-COF/PVDF-HFP, TPDA-COF/PVDF-HFP, and TPBTz-COF/PVDF-HFP) nanofibrous membranes. The PVDF-HFP and COF/PVDF-HFP nanofibrous membranes consist of a nonpolar α-phase, associated with the peak at 764 cm^–1^, and a β-phase, associated with the peaks at 838, 879, 1069, 1177, 1272, and 1402 cm^–1^. Absorption bands corresponding to CH_2_ wagging (1402 cm^–1^), symmetric CF_2_ stretching (1177 cm^–1^), and out-of-plane γ-CF_3_ stretching (1069 cm^–1^) were observed in the PVDF-HFP nanofibrous membranes. A small hump at 1272 cm^–1^ was attributed to symmetric CF_2_ vibrations. The bands at 879 and 838 cm^–1^, assigned to the amorphous β-phase, were also present, indicating the partially amorphous nature of the nanofibrous membranes. Additionally, the absorption peak at 764 cm^–1^ corresponds to the nonpolar α-phase and is attributed to the bending vibration of −CF_2_ groups within the α-phase structure.^30,31^ The FTIR spectra of the COF/PVDF-HFP nanofibrous membranes showed no significant changes with varying COF content compared to the pristine PVDF-HFP membrane.

**Figure 6:**
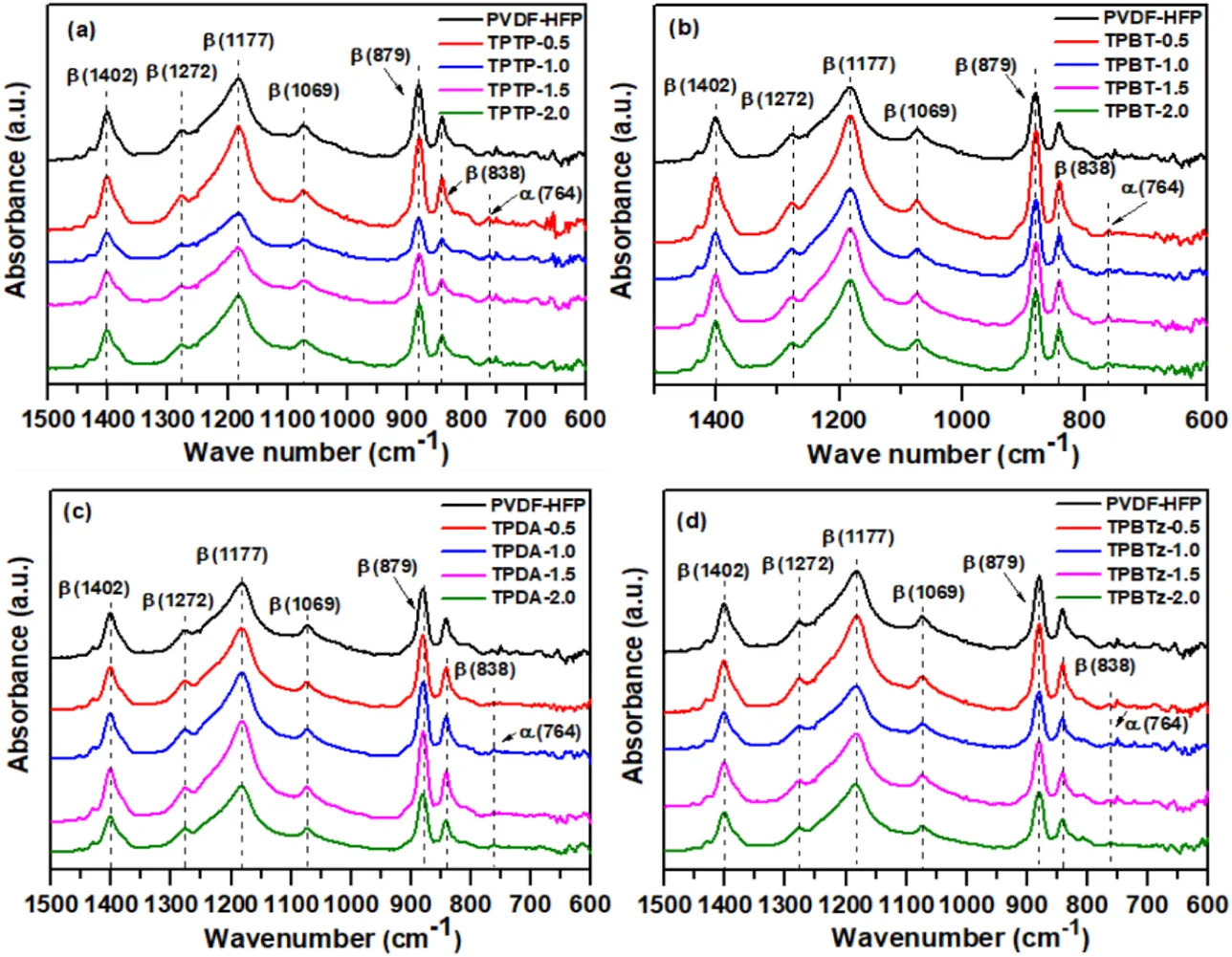
FTIR spectra of the PVDF-HFP and COF/PVDF-HFP nanofibrous films.

The β-phase fraction F­(β) in the COF/PVDF-HFP nanofibrous membranes was calculated using the Lambert–Beer relation:

$$F\left(\beta \right)=\frac{A_{\beta }}{\left(\frac{k_{\beta }}{k_{\alpha }}\right)A_{\alpha }+A_{\beta }}$$

where *A*_α_ and *A*_β_ are the absorbances at 764 cm^–1^ and 840 cm^–1^, respectively, and the absorption coefficients are *k*_α_ = 6.1 × 10^4^ cm^2^·mol^–1^ and *k*_β_ = 7.7 × 10^4^ cm^2^ ·mol^–1^. In Table S2, compared with TPDA-COF and TPBTz-COF, the incorporation of TPTP-COF and TPBT-COF caused a more pronounced decrease in the β-phase content of the PVDF-HFP fibers, particularly for TPTP-COF. The F­(β) value declined markedly with increasing TPTP-COF and TPBT-COF loading. This behavior can be attributed to their large specific surface areas, which lead to strong interactions with the electrospinning solution and disrupt fiber formation, thereby reducing the β-phase fraction. In contrast, TPDA-COF and TPBTz-COF exhibited minimal influence on F­(β) below 1.0 wt %, likely due to their lower specific surface areas and nanorod-like morphologies. However, a noticeable reduction in F­(β) was observed at 1.5 wt %, suggesting that excessive COF loading surpassed the dispersion threshold, promoting aggregation that disturbed the fiber structure and diminished the polar β-phase content. Overall, the effects of TPTP-COF and TPBT-COF on β-phase reduction were more significant than those of TPDA-COF and TPBTz-COF.

The XRD patterns of pristine PVDF-HFP and COF/PVDF-HFP nanofibrous membranes are shown in Figure 7. For the pristine PVDF-HFP membrane, the diffraction peak at 2θ = 18.1° (100) corresponds to the α phase, while the peak at 2θ = 20.1° is assigned to the crystalline β phase. The pristine PVDF-HFP nanofibrous membrane exhibits a stronger β-phase diffraction peak compared to the α-phase peak. Upon incorporating COFs, the intensity of the α-phase peak gradually increased with increasing COF content. Compared with the TPDA-COF/PVDF-HFP and TPBTz-COF/PVDF-HFP samples, a more pronounced enhancement of the α-phase peak intensity was observed for the TPTP-COF/PVDF-HFP and TPBT-COF/PVDF-HFP membranes. Notably, for the TPTP-2.0 sample, the α-phase peak intensity exceeded that of the β phase. This pronounced α-phase enhancement is attributed to the larger specific surface areas of TPTP-COF and TPBT-COF, which induce stronger interactions with the electrospinning solution and disturb fiber formation, thereby suppressing the formation of the β phase in PVDF-HFP.

**Figure 7:**
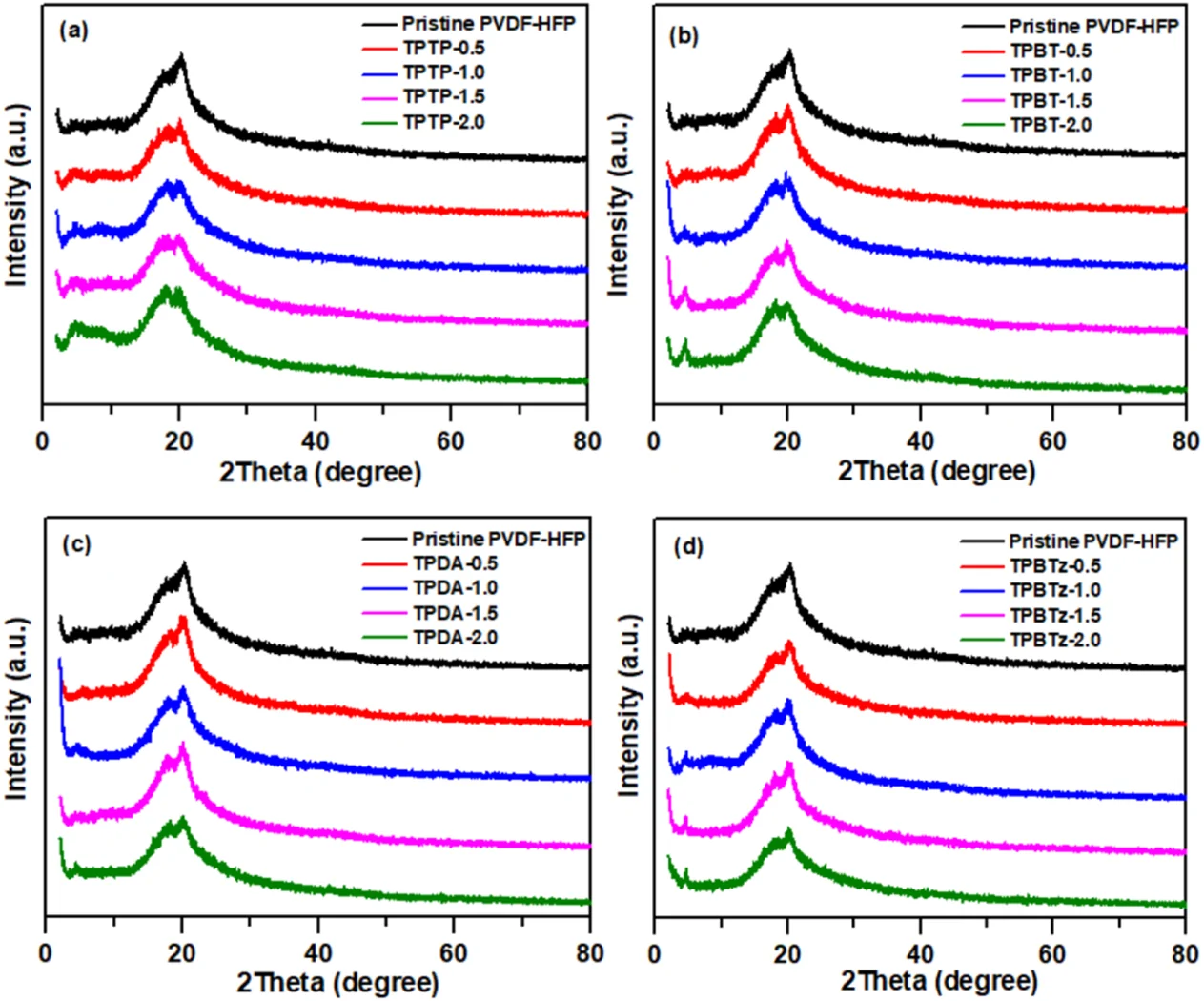
XRD images of the PVDF-HFP and COF/PVDF-HFP nanofibrous films.

The crystalline phases of COF/PVDF-HFP nanofibers with various COF contents were further analyzed. Peak deconvolution in the 2θ range of 10–30° was performed using the FitPeaks (Pro) function in OriginPro (Figures S18–S22). PVDF-HFP primarily exhibits nonpolar α-phase peaks at 2θ = 17.8° (100), 18.2° (020), and 20.1° (110), along with a polar β-phase peak at 2θ = 20.2° (110/200).^32^ The effect of COF incorporation on the crystalline structure of COF/PVDF-HFP nanofibrous membranes is summarized in Table S3. The β (110/200) phase content slightly decreased with increasing COF loading in the TPTP-COF, TPBT-COF, and TPBTz-COF-based PVDF-HFP nanofibrous membranes. This suggests that COF incorporation suppresses β-phase formation while favoring the growth of the α-phase. The encapsulation of COFs within the nanofibers, along with secondary interactions between the highly electronegative N, O, and S atoms of the COFs and the F atoms in PVDF-HFP, contributes to the reduction of β-phase content. Among these, TPTP-COF, possessing a larger specific surface area, resulted in a lower β-phase fraction compared with the TPBT-COF and TPBTz-COF-based membranes. In contrast, the incorporation of TPDA-COF slightly enhanced the β-phase content for the TPDA-0.5 and TPDA-1.0 samples. However, further increasing the TPDA-COF content (TPDA-1.5 and TPDA-2.0) led to a decrease in β-phase content.

The molecular interactions between COFs and PVDF-HFP affect the triboelectric performance of the composite membranes. Hydrogen bonding, dipole–dipole interactions, and van der Waals forces facilitate strong interfacial adhesion and uniform COF dispersion. The interactions between COF and PVDF-HFP results were evidenced by X-ray photoelectron spectroscopy (Figure S23). Compared with the pristine PVDF-HFP membrane, the F 1s peak of PVDF-HFP in the COF/PVDF-HFP composite membranes was slightly influenced by the incorporation of COFs. Moreover, the intensity of the O 1s peak originating from TPDA-COF decreased and exhibited a slight shift toward higher binding energy in the TPDA-1.0 sample. In addition, the S 2p peaks associated with TPBT-COF and TPBTz-COF showed pronounced shifts toward higher binding energies in the TPBT-1.5 and TPBTz-1.5 samples. These results suggest the existence of strong interfacial interactions between the fluorine atoms of PVDF-HFP and the sulfur atoms in the TPBT-1.5 and TPBTz-1.5 composites.

The hydrophobicity of the COF/PVDF-HFP nanofibrous membranes was evaluated by CA measurements, with representative photographs shown in Figure S24. The COF/PVDF-HFP nanofibrous membranes exhibited slightly higher CAs than the pristine PVDF-HFP nanofibrous membrane, indicating enhanced hydrophobicity. The increased hydrophobicity is expected to be favorable for triboelectrification.

### 3.4 Surface Morphologies and Surface Potentials of the TPU and COF/PVDF-HFP Nanofibrous Membranes

In order to investigate the surface morphology and electronic properties of the triboelectric layers, the surface roughness and surface potential of the PVDF-HFP and COF/PVDF-HFP nanofibrous films were characterized using KPFM spectroscopy. The KPFM images of the TPTP-COF, TPBT-COF, TPDA-COF, and TPBTz-COF are shown in Figure S25. The CPD values of TPTP-COF, TPBT-COF, TPDA-COF, and TPBTz-COF were −0.36, 0.15, −0.27, and −0.10 V, corresponding to Φ values of 5.96, 5.45, 5.87, and 5.70 eV, respectively. Compared with TPTP-COF, TPBT-COF and TPBTz-COF exhibit reduced Φ values, which can be attributed to the incorporation of sulfur-containing, electron-donating benzotrithiophene and benzobisthiazole units. In contrast, TPDA-COF shows only a slightly smaller Φ value than TPTP-COF. The introduction of the electron-withdrawing anthraquinone unit does not markedly decrease the Φ value of TPDA-COF, likely due to the competing influences of extended π-conjugation and local charge redistribution within the COF framework. The KPFM images of the PVDF-HFP and COF/PVDF-HFP nanofibrous films are shown in Figure 8, and the corresponding Ra, CPD, and Φ values are summarized in Table 1. Higher surface roughness values were observed for the COF/PVDF-HFP nanofibrous films compared with the pristine PVDF-HFP film, and this increased roughness is beneficial for enhancing charge generation in TENG devices. In addition, the Φ values of the PVDF-HFP, TPTP-1.5, TPBT-1.5, TPDA-1.0, and TPBTz-1.5 nanofibrous films were determined to be 6.25, 6.02, 6.62, 5.97, and 5.76 eV, respectively. Compared with pristine PVDF-HFP, the TPTP-1.5, TPDA-1.0, and TPBTz-1.5 films exhibit lower Φ values, whereas TPBT-1.5 shows a higher Φ value. The decreased Φ values observed for TPTP-1.5, TPDA-1.0, and TPBTz-1.5 can be rationalized by the intrinsically lower Φ values of the incorporated COFs relative to PVDF-HFP. In contrast, the increased Φ value of TPBT-1.5 is likely associated with strong interfacial interactions between the fluorine atoms of PVDF-HFP and the sulfur atoms of TPBT-COF. Such interactions, together with the significant electronegativity difference between PVDF-HFP and the COF component, may induce the formation of an interfacial p–n heterostructure. However, this phenomenon was not observed for TPBTz-1.5, which can likely be attributed to the relatively low specific surface area of TPBTz-COF. The strong interfacial interaction between the fluorine atoms of PVDF-HFP and the sulfur atoms of TPBT-COF was further verified by XPS analysis. The larger Φ difference between the TPU-based positive triboelectric layer and the COF/PVDF-HFP-based negative triboelectric layer promotes stronger charge transfer during the contact–separation cycles, thereby improving the output performance of the TENG.

**Figure 8:**
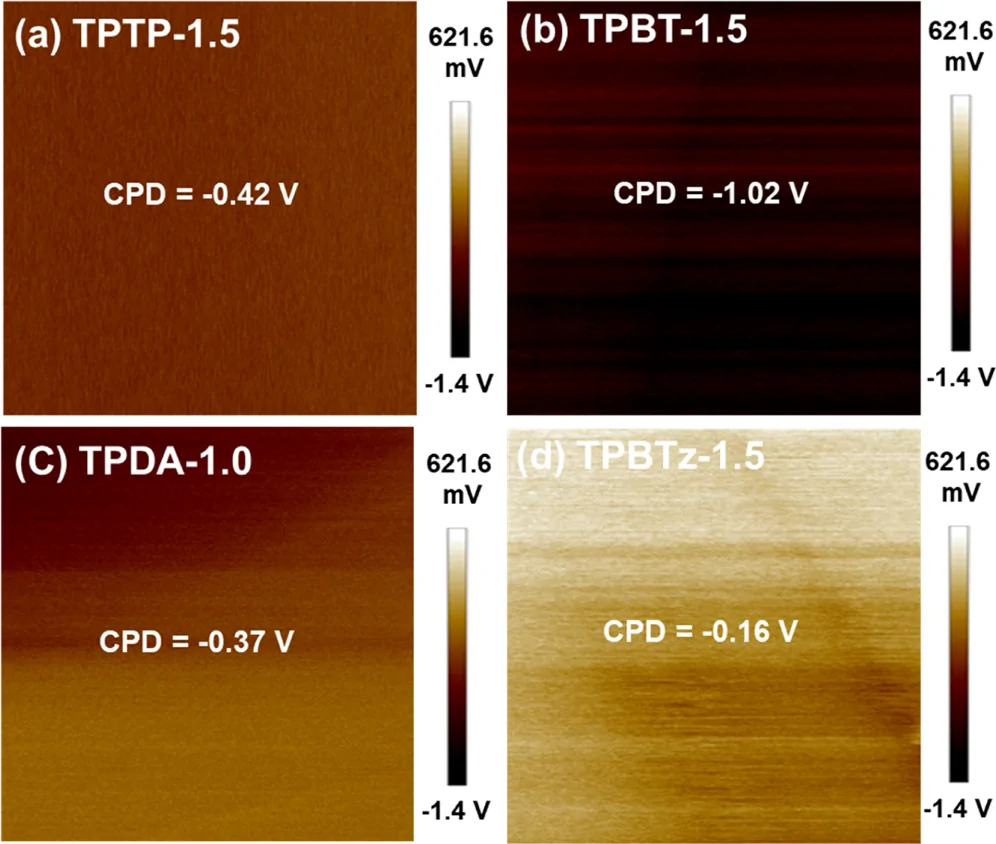
KPFM surface potential maps (1 × 1 μm^2^) of the TPTP-1.5, TPBT-1.5, TPDA-1.0, and TPBTz-1.5 nanofibrous films.

**Table 1:** Surface Roughness (Ra), Contact Potential Difference (CPD), and Φ of PVDF-HFP and COF/PVDF-HFP Nanofibrous Membranes

Sample	Ra (nm)	V_CPD_ (V)	Φ (eV)
PVDF-HFP	7.48 ± 0.23	–0.65 ± 0.12	6.25 ± 0.12
TPTP-1.5	11.52 ± 0.25	–0.42 ± 0.11	6.02 ± 0.11
TPBT-1.5	8.96 ± 0.30	–1.02 ± 0.13	6.62 ± 0.13
TPDA-1.0	18.61 ± 0.24	–0.37 ± 0.08	5.97 ± 0.08
TPBTz-1.5	10.10 ± 0.25	-016 ± 0.10	5.76 ± 0.10

### 3.5 Dielectric Characteristics of the COF/PVDF-HFP Nanofibrous Membranes

The surface charge density of the triboelectric layer is positively correlated with its dielectric constant, indicating that increasing the dielectric constant can effectively enhance the triboelectric performance of the TENG.^33,34^ The dielectric constant of the PVDF-HFP membrane is higher than that of the TPU film (Figure 9). Moreover, the dielectric constant of the PVDF-HFP membrane further increased upon the incorporation of the COF. High capacitance values of TPTP-COF, TPBT-COF, and TPDA-COF have been reported in the literature,^12,16^ implying that these COFs possess high dielectric constants. A high dielectric constant in the COF promotes the generation of a greater surface charge density on the triboelectric layer during contact electrification. In addition, these COFs contain nitrogen, oxygen, and sulfur atoms with lone-pair electrons. When used as additives in PVDF-HFP to form composite materials, they induce interfacial polarization under an applied electric field, thereby increasing the dielectric constant of the COF/PVDF-HFP composite films. This enhanced dielectric property facilitates surface charge accumulation, resulting in higher electrostatic output. Among the composite films, TPBT-1.5 exhibits a slightly higher dielectric constant than TPTP-1.5, while both TPBT-1.5 and TPTP-1.5 show higher dielectric constants than TPDA-1.0 and TPBTz-1.5. The increased specific surface area of TPTP-COF and TPBT-COF enlarges the effective contact area within the PVDF-HFP fibers, thereby creating more charge-trapping sites that can capture electrons and generate a higher surface charge density,^6,35^ In addition to the intrinsic properties of the COF, the influence of the COF on the dielectric constant of the composite membranes is closely related to its specific surface area. The lower specific surface areas of TPDA-COF and TPBTz-COF diminish their ability to enhance the dielectric constant of the COF/PVDF-HFP composite membranes.

**Figure 9:**
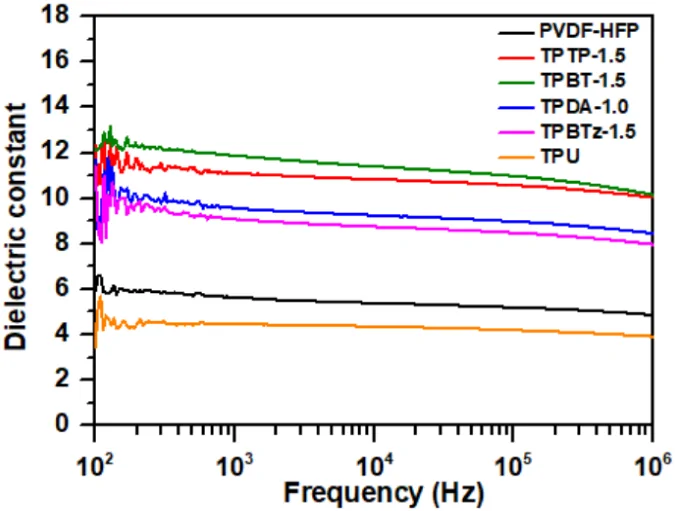
Dielectric constant versus frequency of the COF/PVDF-HFP membranes.

### 3.6 Electrical Characteristics of the COF/PVDF-HFP Nanofibrous Membrane-Based TENGs

The energy generated by a TENG is positively correlated with the amount of charge that the friction materials can capture and store. Typically, factors such as the material properties, the effective contact area between the friction layers, the contact frequency, and the externally applied mechanical force all influence the output performance of the TENG. As shown in Figure S26, the contact frequency was fixed (5 Hz), and the effects of different applied forces (20–70 N) on the *V*_OC_ of the COF/PVDF-HFP-based TENGs were measured. The *V*_OC_ increased with increasing applied force. This is mainly because, under applied force, the friction layer is compressed, thereby increasing the effective contact area and enhancing the amount of accumulated charge. Additionally, higher applied force increases the elastic deformation of the friction layer, which further increases the capacitance of the COF/PVDF-HFP TENG and enhances charge storage. Applying greater force also promotes the generation of more surface triboelectric charges, thereby improving the electrical output. However, when the applied force reaches 70 N, it exceeds the mechanical tolerance of the nanofibrous membranes, causing surface damage, which results in a decrease in overall electrical performance.

*V*_OC_ and I_SC_ of the TPTP-1.5, TPBT-1.5, TPDA-1.0, and TPBTz-1.5 TENGs measured at 60 N under various applied frequencies are shown in Figure 10. Higher contact frequencies led to increased *V*_OC_ and I_SC_ values. This enhancement is primarily attributed to the higher contact–separation frequencies accelerating the rate of electron transfer, allowing charges to move more rapidly.^7^ The highest *V*_OC_ and I_SC_ values were observed for all the COF/PVDF-HFP nanofibrous membrane-based TENGs at a frequency of 5 Hz under an applied force of 60 N.

**Figure 10:**
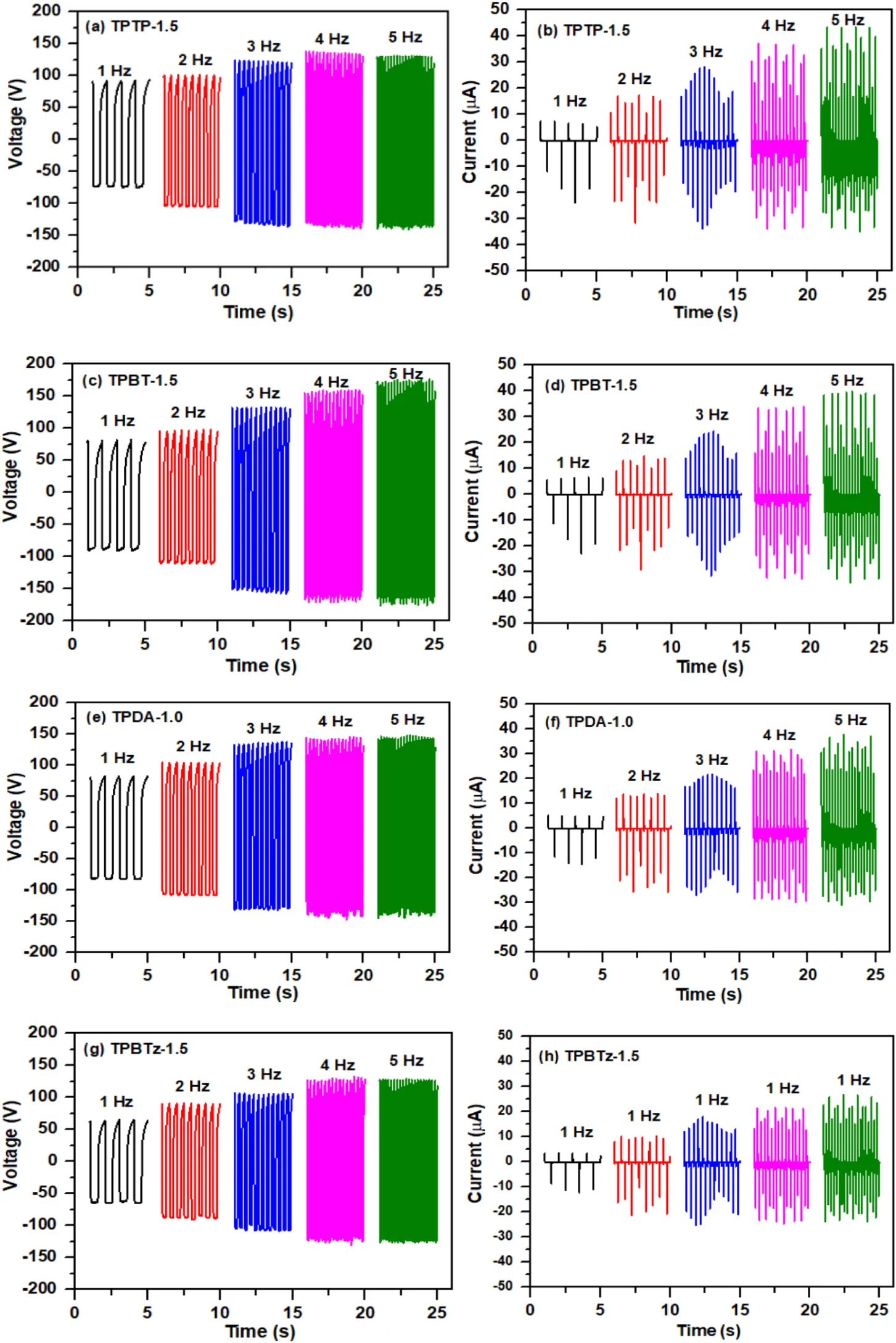
*V*_OC_ and I_SC_ of the TENGs measured at 60 N under various applied frequencies [(a), (b): TPTP-1.5 TENG: (c), (d): TPBT-1.5 TENG; (e), (f): TPDA-1.0 TENG; (g), (h): TPBTz-1.5 TENG].

The COF content effect on the *V*_OC_ and I_SC_ of the COF/PVDF-HFP based TENGs are shown in Figure 11. Compared to the pristine PVDF-HFP-based TENG, the COF/PVDF-HFP-based TENGs exhibited higher *V*_OC_ and I_SC_ values. The pristine PVDF-HFP-based TENG showed *V*_OC_ and I_SC_ values of 207.2 V and 23.2 μA, respectively. Both *V*_OC_ and I_SC_ increased significantly with increasing COF content in the COF/PVDF-HFP-based TENGs. The highest *V*_OC_ and I_SC_ values were obtained at a COF loading of 1.5 wt %, while the optimal content for the TPDA-COF/PVDF-HFP-based TENGs was 1.0 wt %. The maximum *V*_OC_ values of the TPTP-1.5, TPBT-1.5, TPDA-1.0, and TPBTz-1.5 TENGs were 276.8, 338.2, 271.0, and 250.0 V, respectively, while the corresponding I_SC_ values were 76.9, 74.0, 66.0, and 50.5 μA. The polar COFs, when used as additives in PVDF-HFP to form composite materials, induce interfacial polarization under an applied electric field, thereby increasing the dielectric constant and promoting surface charge accumulation, which leads to higher electrostatic output.^31,34^ Excessive COF content led to a decrease in the triboelectric performance of the COF/PVDF-HFP-based TENGs, as observed for the 2.0 wt % COF sample. This reduction may be attributed to the excessive COF content exceeding the percolation threshold, which weakens the interfacial polarization effects and lowers both the dielectric constant and capacitance of the nanofibrous membrane, thereby diminishing the overall triboelectric output efficiency.^23,36^ In addition, the *V*_OC_ and I_SC_ values of the TPTP-COF/PVDF-HFP- and TPBT-COF/PVDF-HFP-based TENGs were considerably higher than those of the TPDA-COF/PVDF-HFP- and TPBTz-COF/PVDF-HFP-based counterparts. This enhancement mainly arose from the larger specific surface areas of TPTP-COF and TPBT-COF, which strengthened interfacial interactions with the PVDF-HFP matrix. Moreover, the large electronegativity difference between PVDF-HFP and TPBT-COF gives rise to an interfacial p–n heterostructure. The resulting polarity mismatch at the interface facilitates charge transfer during frictional contact, thereby enhancing the charge generation of the TPBT-COF/PVDF-HFP-based TENGs.

**Figure 11:**
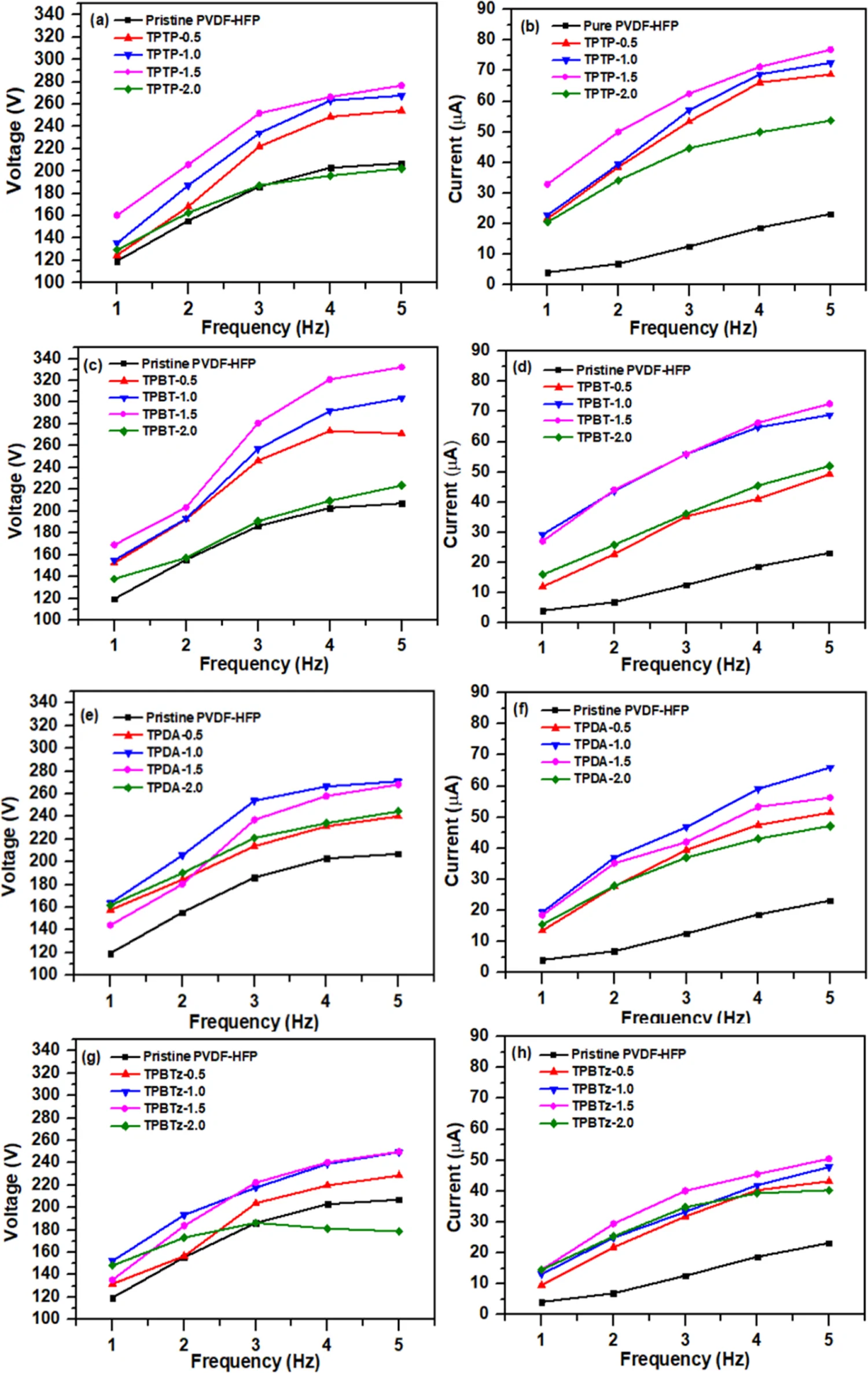
*V*_OC_ and I_SC_ of the TENGs measured at 60 N under various applied frequencies [(a), (b): TPTP-COF TENG; (c), (d): TPBT-COF TENG; (e), (f): TPDA-COF TENG; (g), (h): TPBTz-COF TENG].

The *V*_OC_, I_SC_, and transferred charge of the pristine PVDF-HFP and COF/PVDF-HFP-based TENGs were measured at 5 Hz under an applied force of 60 N (Figure 12). The incorporation of COFs significantly improved the electrical properties of the PVDF-HFP-based TENGs. Among them, the *V*_OC_ and transferred charge values of the TPBT-1.5 TENG were much higher than those of the TPTP-1.5, TPDA-1.0, and TPBTz-1.5 TENGs. In addition, the Isc values of the TPTP-1.5 and TPBT-1.5 TENGs were higher than those of the TPDA-1.0 and TPBTz-1.5 TENGs. The optimized TPBT-COF/PVDF-HFP membrane based TPBT-1.5 TENG achieved maximum *V*_OC_, I_SC_, and transferred charge of 338.2 V, 74.0 μA, and 127.1 nC, respectively.

**Figure 12:**
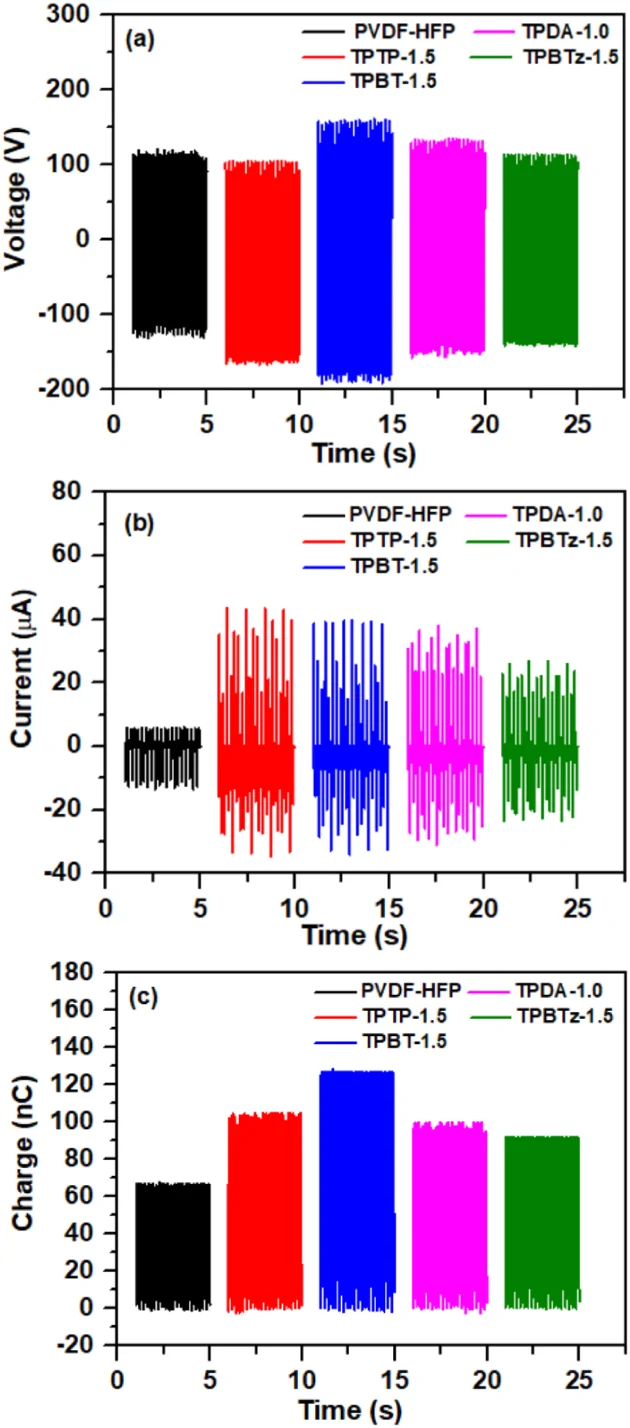
Electric properties of the PVDF-HFP and COF/PVDF-HFP composite films based TENGs measured at 5 Hz under applied force of 60 N [(a) *V*_OC_, (b) I_SC_, and (c) transfer charge.

To better understand the maximum potential distribution during TENG operation, the electrostatic potential distribution between the positive and negative triboelectric layers at a separation distance of 10 mm was simulated using COMSOL Multiphysics, as shown in Figure S27. The simulation parameters are summarized in Table S4. In the initial contact state, the TPBT-1.5 TENG was in electrostatic equilibrium, resulting in a negligible potential difference between the two triboelectric layers. As the separation distance increased to 10 mm, the simulated *V*_OC_ reached 338.0 V. The simulated *V*_OC_ was in good agreement with the experimentally measured value, confirming that the vertical contact–separation motion of the triboelectric layers effectively converts mechanical energy into electrical energy.

At an applied force of 60 N and a frequency of 5 Hz, the COF/PVDF-HFP-based TENGs were connected to various external load resistances, and their electrical outputs were measured over a resistance range from 1 Ω to 1 GΩ. As shown in Figure 13a and b, the *V*_OC_ increased with increasing external load resistance, whereas the I_SC_ showed a decreasing trend. Among the devices, the TPBT-1.5 TENG exhibited both the highest *V*_OC_ and the highest I_SC_, while the TPBTz-1.5 TENG showed the lowest values compared with the other COF/PVDF-HFP-based TENGs. The power density of the TPBT-1.5 TENG was higher than those of the TPTP-1.5, TPDA-1.0, and TPBTz-1.5 devices. A maximum output power density of 16.76 W/m^2^ was achieved at a load resistance of 50.0 MΩ for the TPBT-1.5 TENG. As shown in Figure 13c and d, the output voltages of the TPTP-1.5- and TPBT-1.5-based TENGs were monitored over 20,000 continuous contact–separation cycles. The *V*_OC_ values of the TPTP-1.5 and TPBT-1.5 devices remained at approximately 250 and 325 V, respectively, even after repeated impacts, demonstrating the excellent cycling durability and stability of these COF/PVDF-HFP-based TENGs.

**Figure 13:**
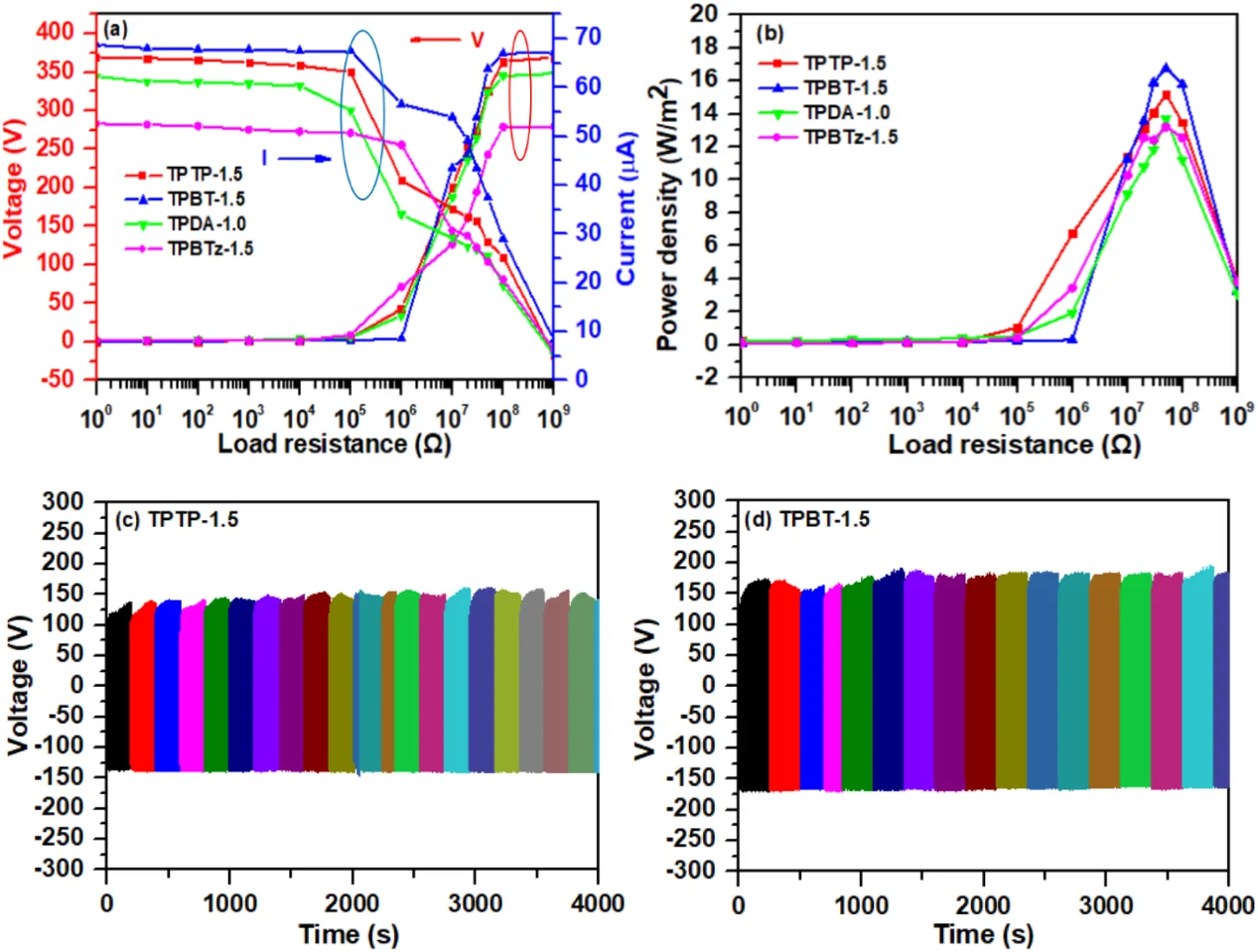
(a) Output and voltage current of TENG with various external load resistances, (b) dependence of output power on load resistances, and (c, d) durability test of the TPTP-1.5 and TPBT-1.5 based TENGs under 60 N pressure and 5 Hz for 20000 cycles.

To facilitate a comparison between the triboelectric performance of this work and that reported in the literature using COF materials for triboelectric nanogenerators, the output voltage, current density, and power density of COF-based triboelectric layers reported in previous studies are summarized in Table 2. As summarized in Table 2, most previous studies reported that COF nanoparticles were sprayed and adsorbed onto the surfaces of Cu or Ni foils to serve as the positive triboelectric layer in TENGs.^14,23,24,26–28^ Although high *V*_OC_ values were observed for these literature-reported TENGs (Table 2), the output I_SC_ and power density of the TPBT-1.5-based TENG were found to be superior to those of the previously reported devices.

**Table 2:** Output Performance Characteristics of the Optimized TENG in This Work Compared with Previously Reported COF-Based TENGs

Friction layer	*V*_OC_ (V)	I_SC_ (μA/cm^2^)	Power density (W/m^2^)	Ref.
TPBT-COF/PVDF-HFP	338.2	12.30	16.76	This study
Triazine-COF/Cu	70.0	0.10	9.5 × 10^–4^	^13^
Tp-TFAB-COF/Ni	177.8	2.60	8.24	^21^
TPB-DBBA-COF/Cu	416.0	1.75	5.29	^22^
TFD-DP-COF/Cu	1000.0	3.29	0.034	^24^
TpPa-S-COF/Cu	112.7	4.40	0.59	^26^

### 3.7 Application of the COF/PVDF-HFP-Based TENG

To enable the TENG to drive wearable devices and operate continuously, a bridge rectifier was used as an AC-to-DC converter and connected to a capacitor as an energy storage unit, forming a self-powered system (Figure S28). As shown in Figure S29, the COF/PVDF-HFP-based TENGs were used to charge commercial electrolytic capacitors with capacitances of 1, 10, and 100 μF under an applied force of 60 N and a contact frequency of 5 Hz. The 1 μF capacitor was charged to 30 V within approximately 80 s by all TENGs except the TPBTz-1.5 TENG, which exhibited a slightly lower charging rate than the other devices. As the capacitance increased from 1 to 100 μF, the charging rate gradually decreased for all TENGs.

To demonstrate the practical applicability of the high electrical output of the TENG for powering LEDs, the TPBT-1.5 TENG was connected to a bridge rectifier and then linked in series with 100 commercial LEDs, as shown in Figure 14a. Under an applied force of 60 N and a contact frequency of 5 Hz, all 100 LEDs were simultaneously illuminated, confirming that the TENG can effectively convert mechanical energy into electrical energy to drive electronic components. Furthermore, two TPBT-1.5 TENGs connected in parallel and another two connected in series were mounted on a custom-made insole (Figure S30) and placed inside a shoe. The output wires were routed to an external circuit board mounted on the shoe (Figure 14b). A researcher weighing 70 kg wore the shoe and performed jogging to generate electrical energy, which was then stored in a connected capacitor and subsequently used to power an LED (Figure 14c,d). During the experiment, it was found that after approximately 3 min of running, the capacitor voltage reached 1.9 V. This study confirms that the developed COF/PVDF-HFP TENG, when installed in an insole, can be applied as a walk-induced energy-harvesting device for athletes or hikers, serving as a power source for wearable electronic devices.

**Figure 14:**
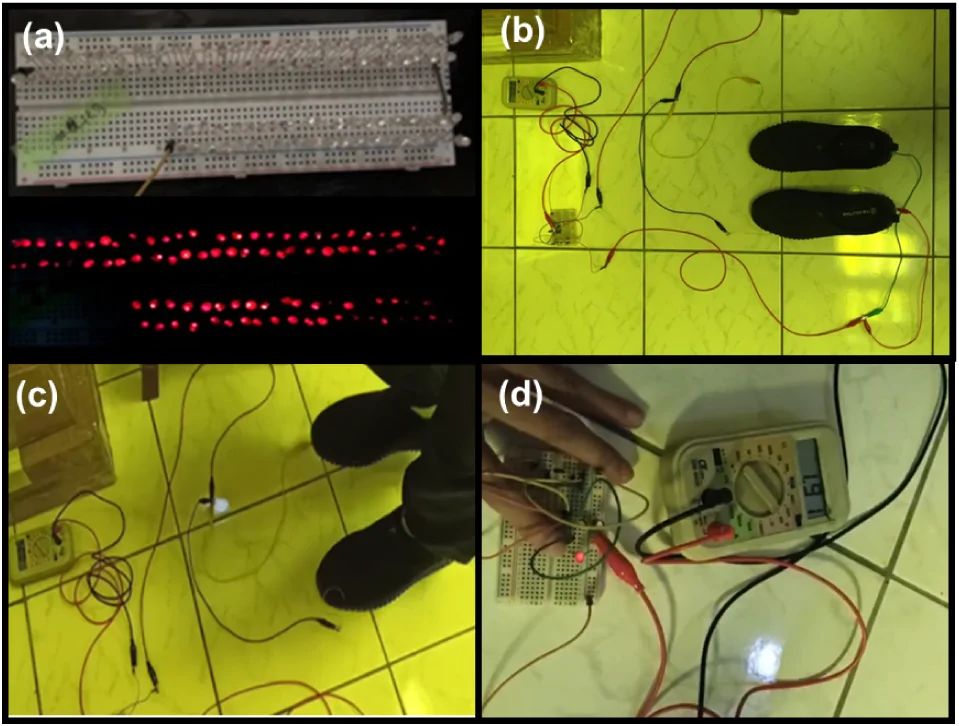
TPBT-1.5-based TENG for practical energy-harvesting demonstrations: (a) powering 100 commercial LEDs; (b) TENG devices installed in an insole; and (c, d) a 70-kg researcher wearing the shoe and jogging to generate electrical energy, which was subsequently used to power an LED.

## 4 Conclusion

The COF/PVDF-HFP composite electrospun nanofibrous membranes were developed as the negative friction layer of the TENG. SEM images revealed that the COF/PVDF-HFP nanofibers exhibited well-defined unidirectional alignment. The fiber diameters were not changed significantly as the COF content increased. EDX analyses further confirmed the uniform distribution of COF particles throughout the PVDF-HFP nanofibrous matrix. The COF/PVDF-HFP-based TENG exhibited markedly improved electrical performance compared with the pristine PVDF-HFP-based device. Incorporating high-dielectric constant COFs into PVDF-HFP induces interfacial polarization under an applied electric field, which increases the dielectric constant and enlarges the surface potential difference relative to the positive friction layer, ultimately improving charge generation during contact–separation cycles. The TPTP-COF- and TPBT-COF-based TENGs generated higher triboelectric output compared to the TPDA-COF- and TPBTz-COF-based devices, owing to their larger specific surface areas, higher dielectric constants, and more efficient surface charge generation. Under operating conditions of 60 N applied force at 5 Hz, the COF/PVDF-HFP-based TENG successfully powered LEDs, while maintaining stable Voc performance over 20,000 contact–separation cycles. The developed COF/PVDF-HFP TENG, when installed in an insole, can be applied as a walk-induced energy-harvesting device for athletes or hikers, serving as a power source for wearable electronic devices.

## Supplementary Material



## Notes

SEM images and particle size distributions of the COFs; SEM images and fiber diameter distribution of COF/PVDF-HFP nanofibrous membranes; TEM images and EDX images of COF/PVDF-HFP nanofibrous membranes; XRD patterns, XPS spectra, and KPFM surface potential maps of COF/PVDF-HFP nanofibrous membranes; *V*_OC_ of the COF/PVDF-HFP based TENGs; charging of commercial capacitors with different capacitances by the TPBT-1.5-based TENG; power-generating insole assembled by connecting four TPBT-1.5 TENGs (PDF)
