# Supplementary material for: High-Performance Triboelectric Nanogenerators Based on Triazine-Based Covalent Organic Framework/PVDF-HFP Composite Nanofibrous Membranes for Wearable Electronics
Source: ACS Polym Au. 2026 Jul 27;6(5):1389–402. doi: 10.1021/acspolymersau.6c00106 (PMC13673881; doi:10.1021/acspolymersau.6c00106)
Supplement: Supplementary file 1 [file lg6c00106_si_001.pdf]

Supporting Information

High-Performance Triboelectric Nanogenerators Based on Triazine-Based Covalent Organic Framework/PVDF-HFP Composite Nanofibrous Membranes for Wearable Electronics

Ying-Hao Pan[1], Hsin-Jung Kan[1], Pei-Cih Hu[1], Hongta Yang[1], and Rong-Ho Lee[1,2]*

1. Department of Chemical Engineering, National Chung Hsing University, Taichung 402, Taiwan.
2. Department of Chemical Engineering and Materials Science, Yuan Ze University, Taoyuan City 320, Taiwan.

*To whom correspondence should be addressed

Rong-Ho Lee—e-mail: rhl@dragon.nchu.edu.tw; tel.: +886-4-22854308; fax: +886-4-22854734

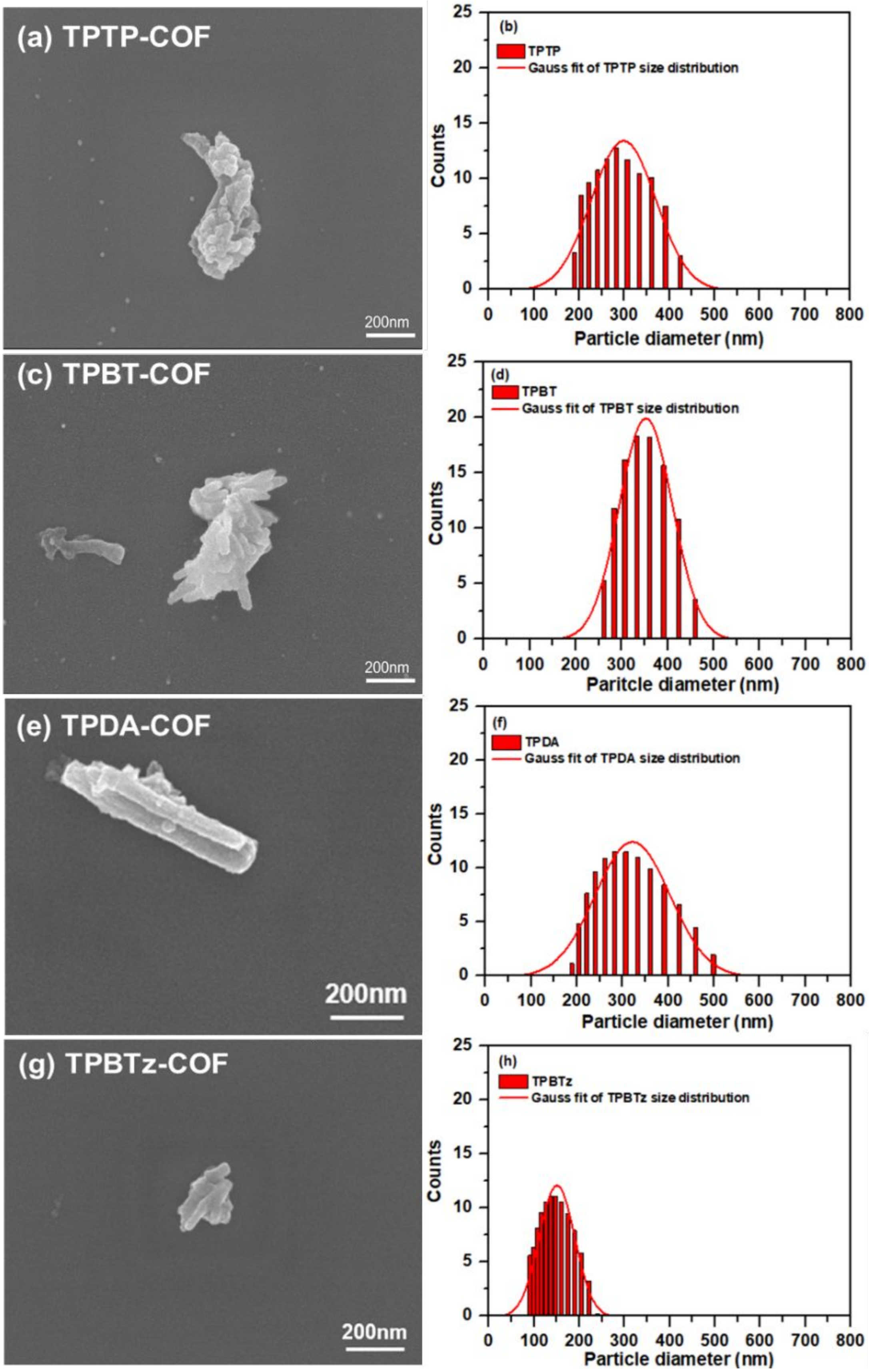

Figure S1. SEM images and particle size distributions of the COFs: (a,b) TPTP-COF; (c,d) TPBT-COF; (e,f) TPDA-COF; (g,h) TPBTz-COF.

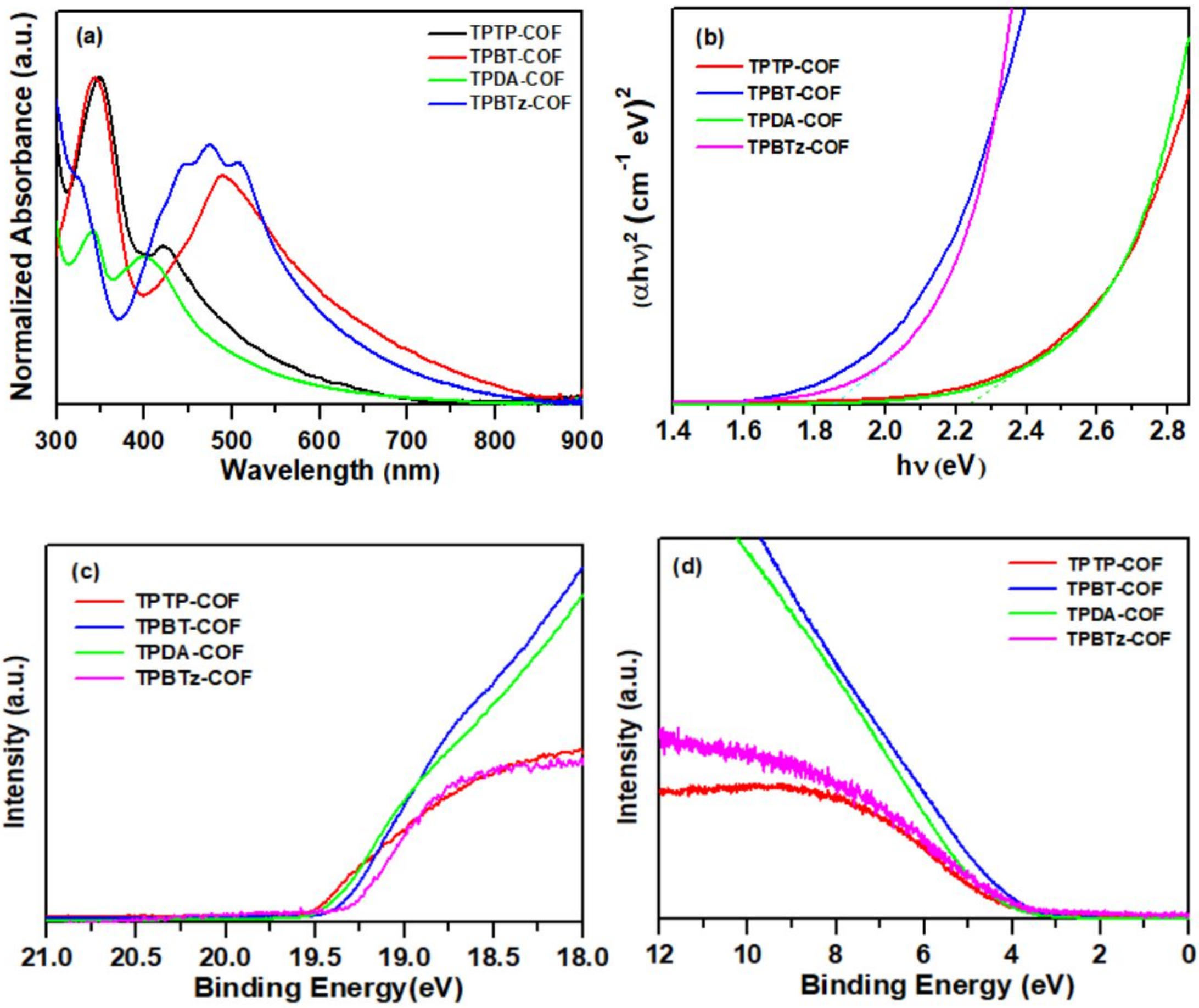

Figure S2. (a) UV–vis absorption spectra, (b) Tauc plots, (c) UPS secondary electron cutoff (E_{cutoff}), and (d) UPS onset energy (E_{onset}) used to determine the electronic energy levels of the COF materials.

Table S1. Energy levels relative to the vacuum level of the COF materials estimated from UPS spectra.

COFs	E_{cutoff} (eV)	E_{onset} (eV)	WF (eV)	E_g (eV)	CBM (eV)	VBM (eV)
TPTP-COF	19.50	4.10	1.72	2.23	-5.82	-3.59
TPBT-COF	19.40	3.90	1.82	1.75	-5.72	-3.97
TPDA-COF	19.45	4.00	1.77	2.24	-5.77	-3.53
TPBTz-COF	19.37	3.95	1.85	1.85	-5.80	-3.95
BTDA-COP	19.38	3.98	1.84	2.35	-5.82	-3.47

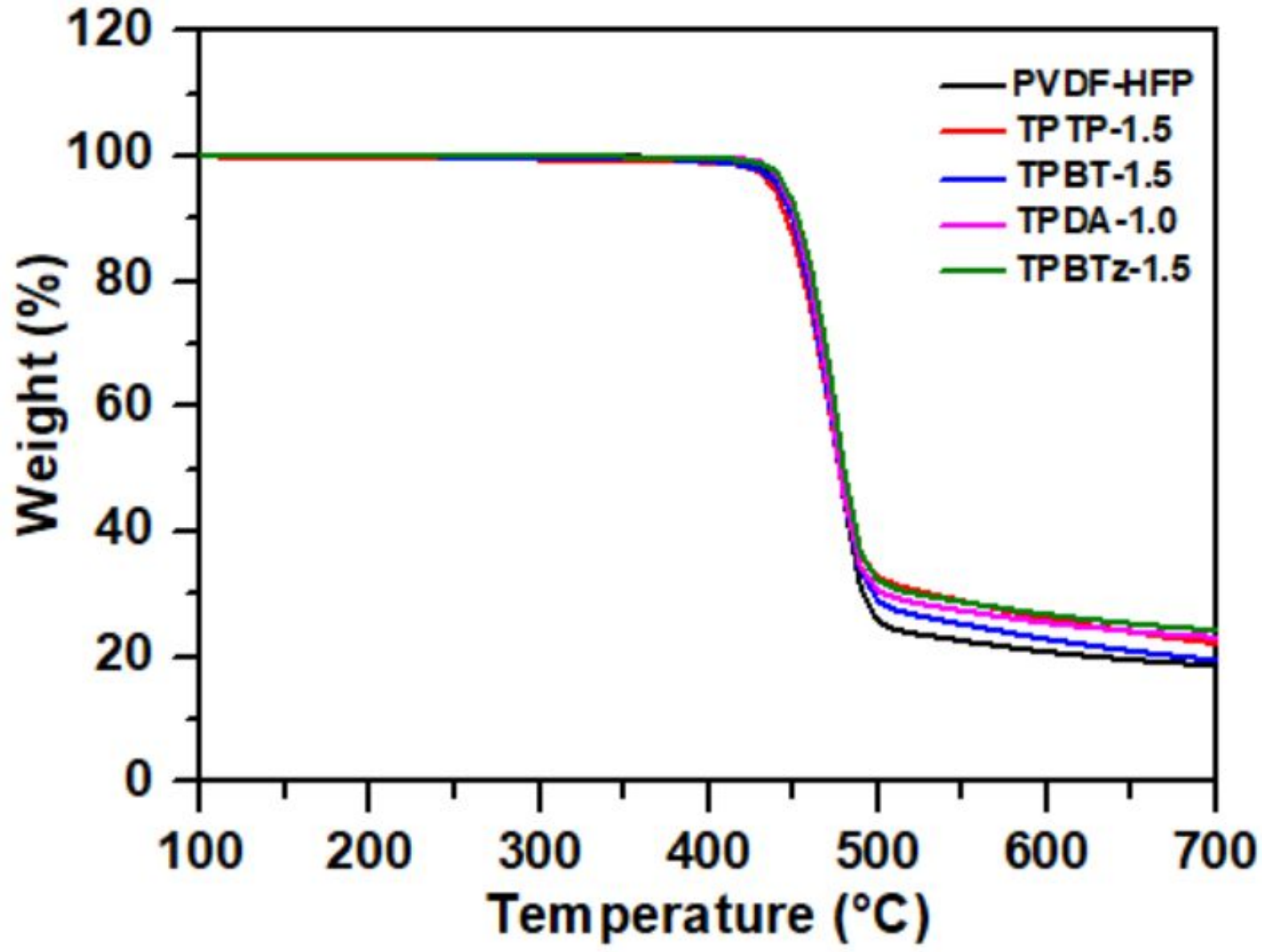

Figure S3. TGA curves of the COF/PVDF-HFP nanofibrous membranes.

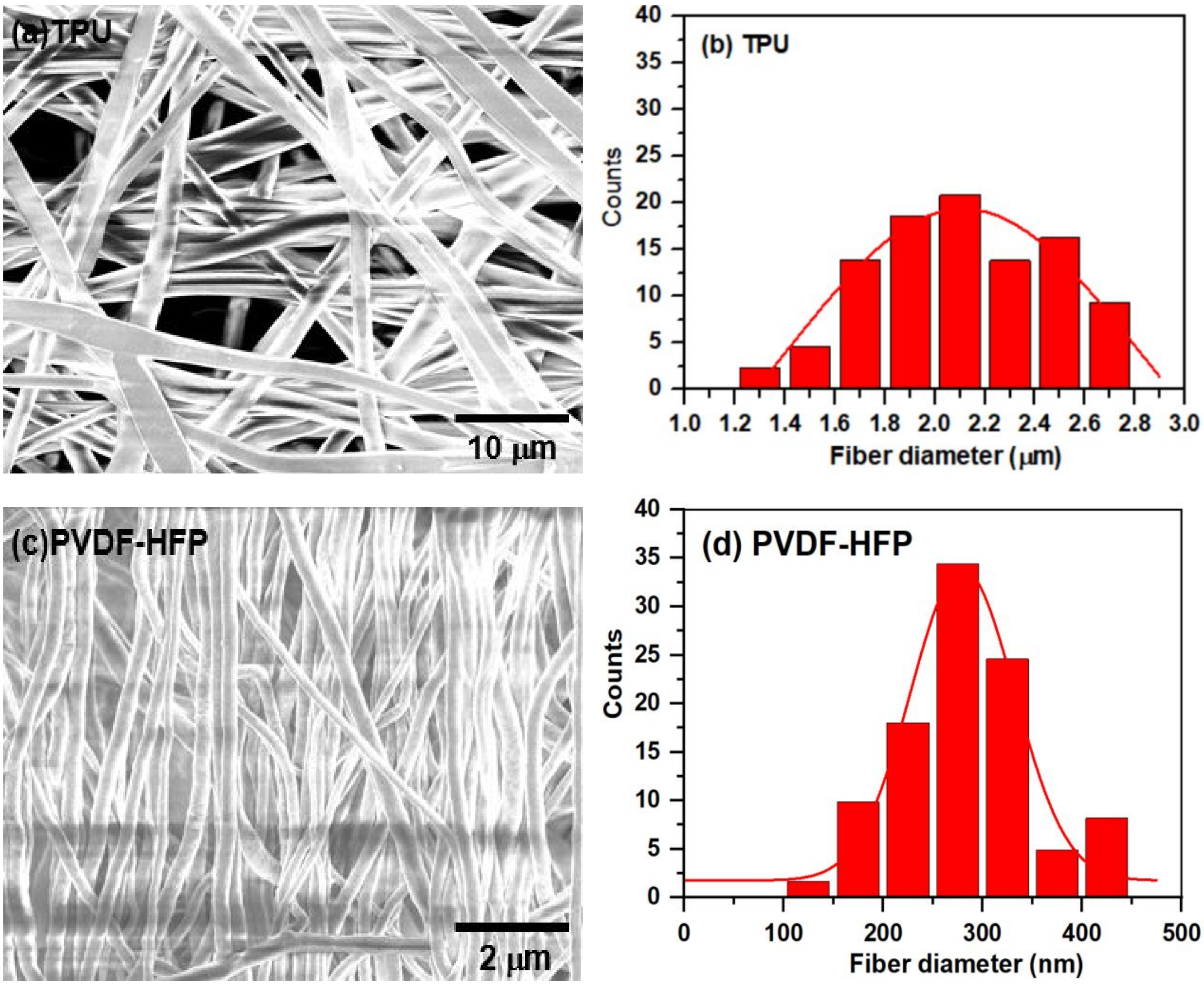

Figure S4. SEM images and fiber diameter distribution of (a,b) TPU and (c,d) PVDF-HFP nanofibrous membranes.

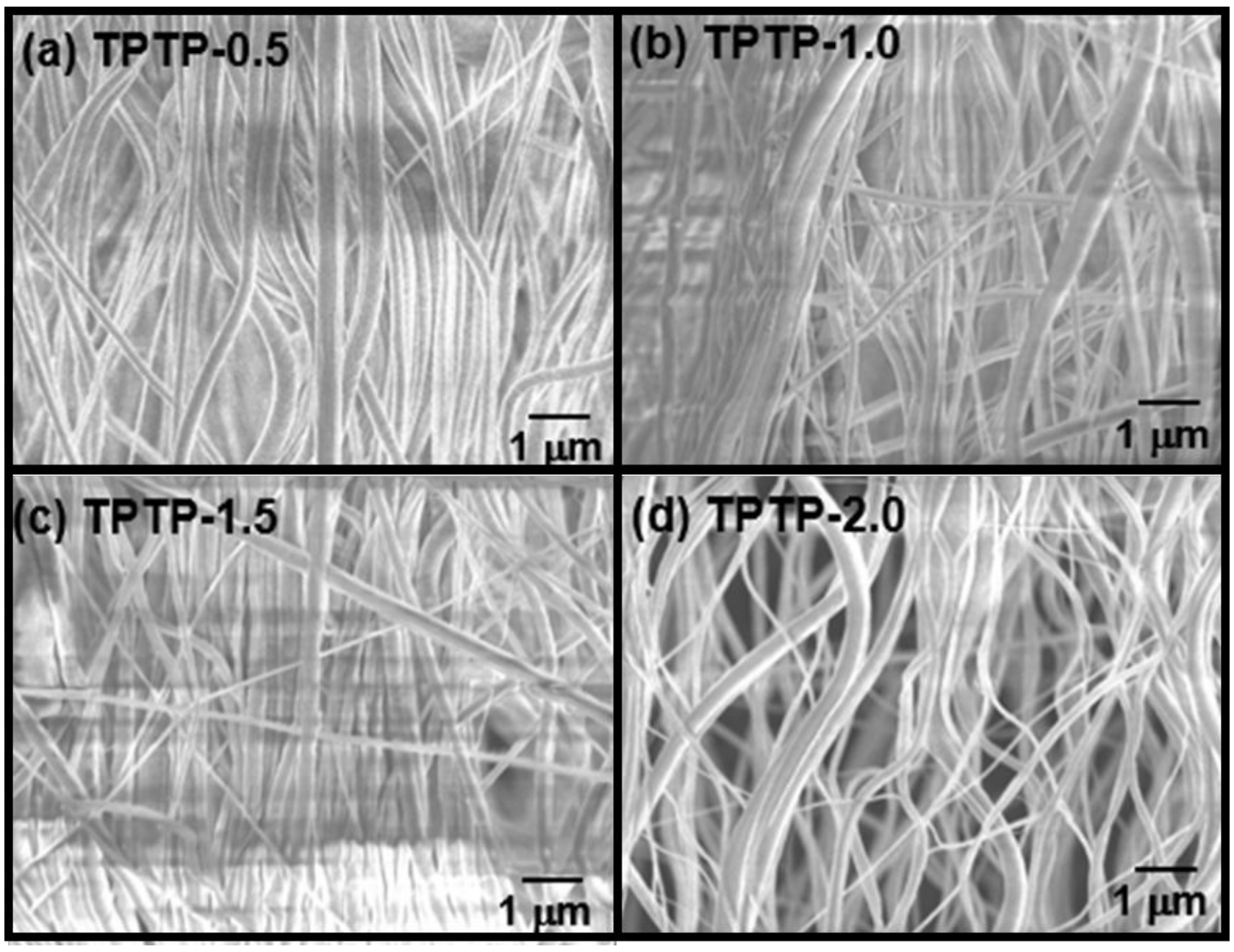

Figure S5. SEM images of the TPTP-based PVDF-HFP nanofibrous membranes.

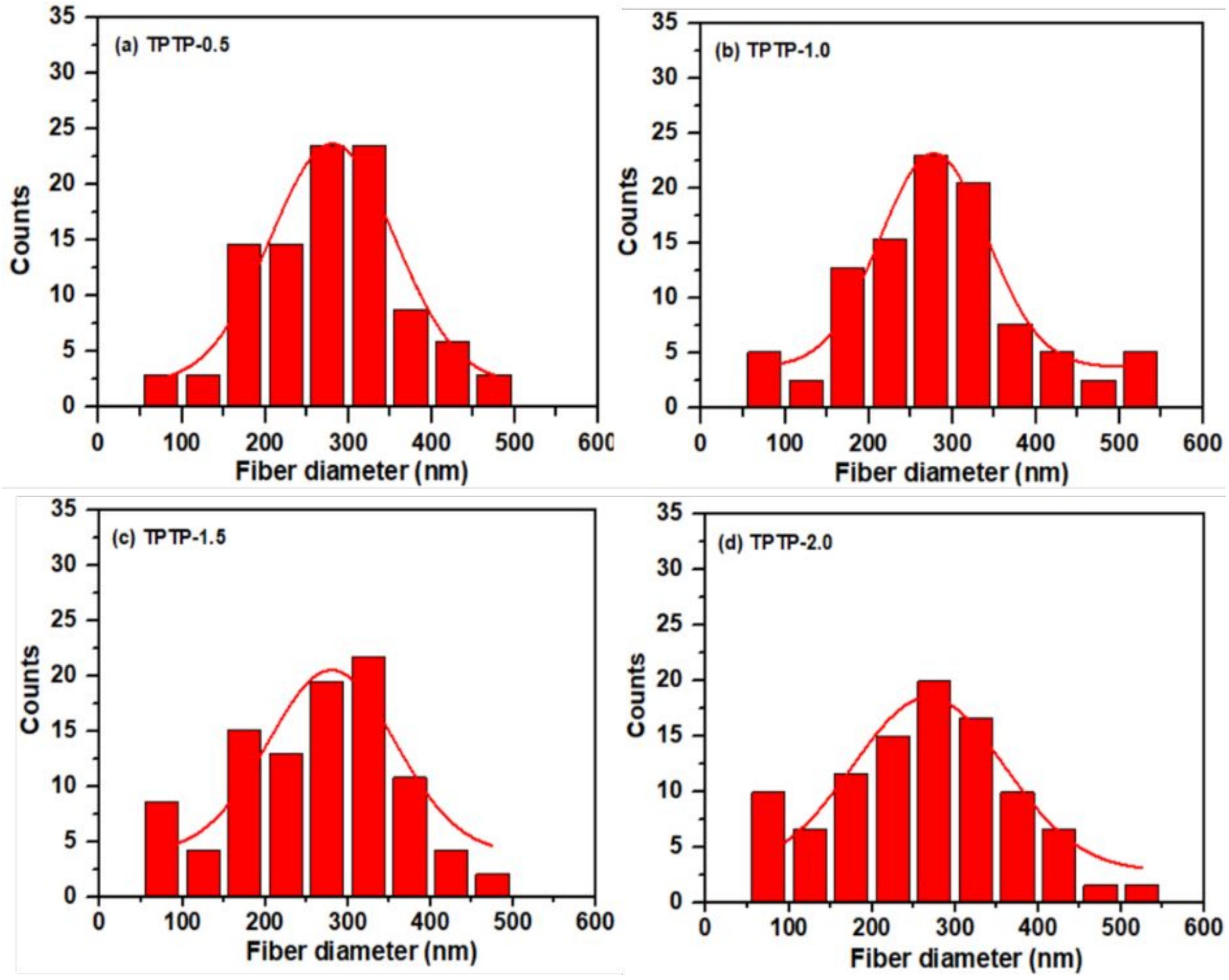

Figure 6. Fiber diameter distribution of the TPTP-based PVDF-HFP nanofibrous membranes.

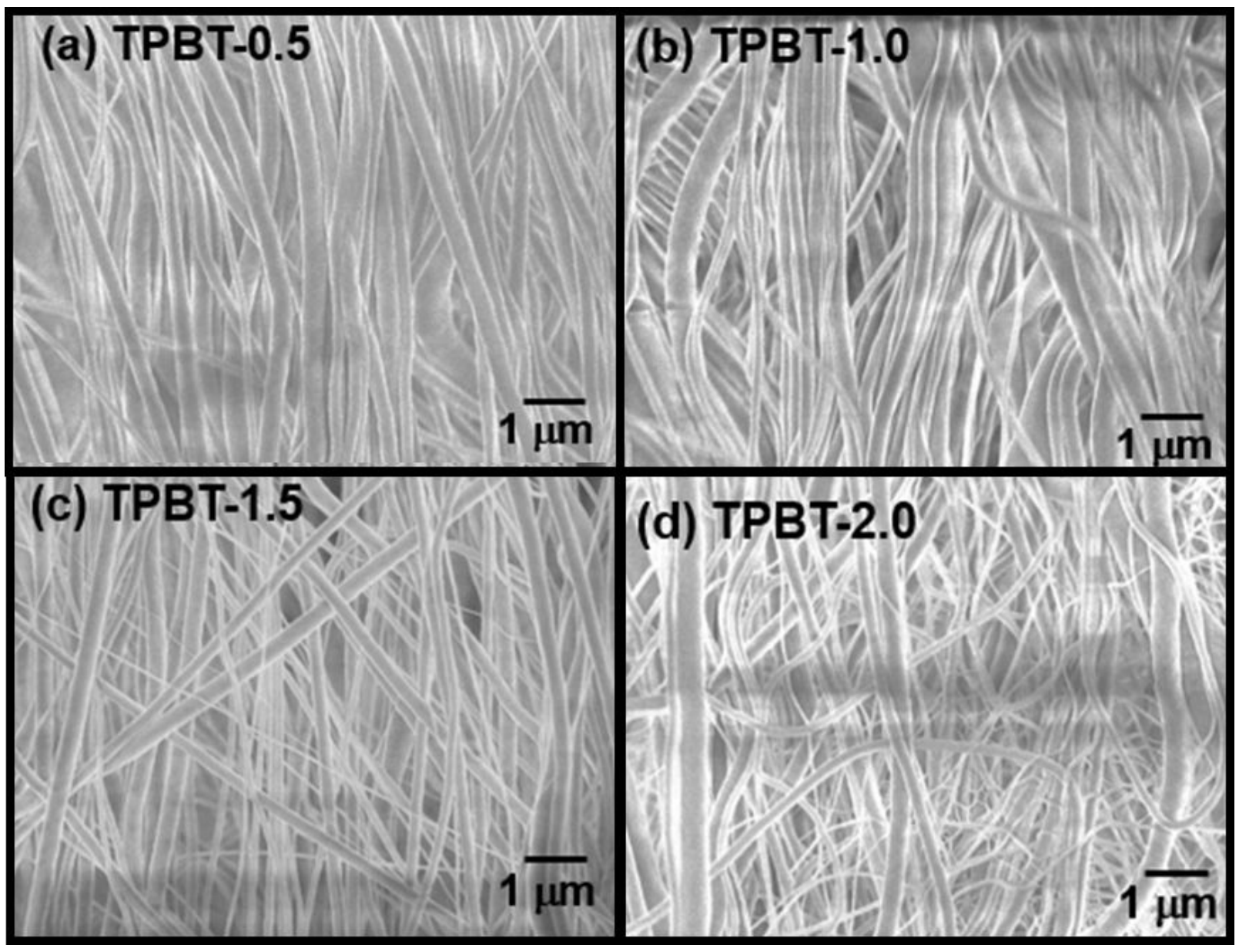

Figure S7. SEM images of the TPBT-based PVDF-HFP nanofibrous membranes.

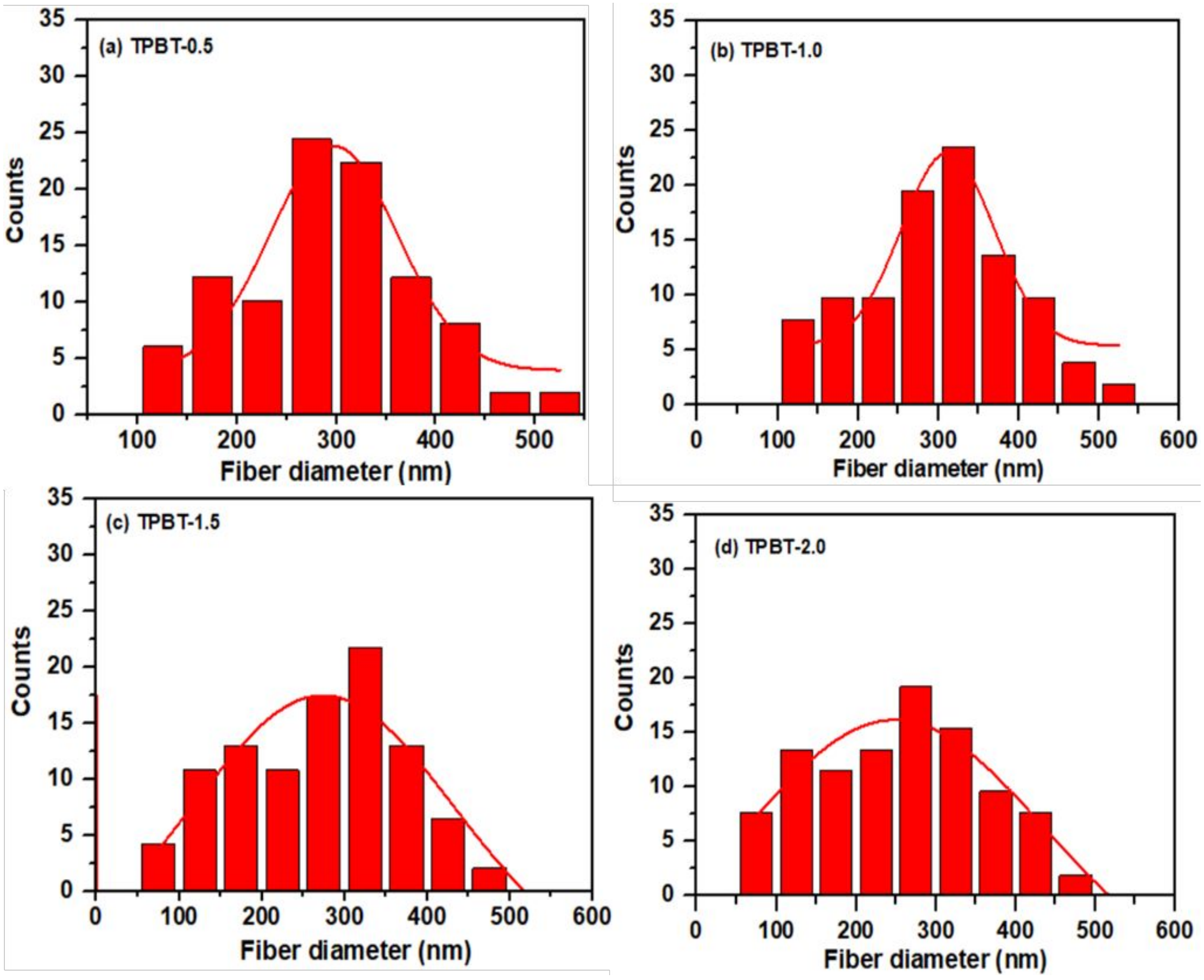

Figure S8. Fiber diameter distribution of the TPBT-based PVDF-HFP nanofibrous membranes.

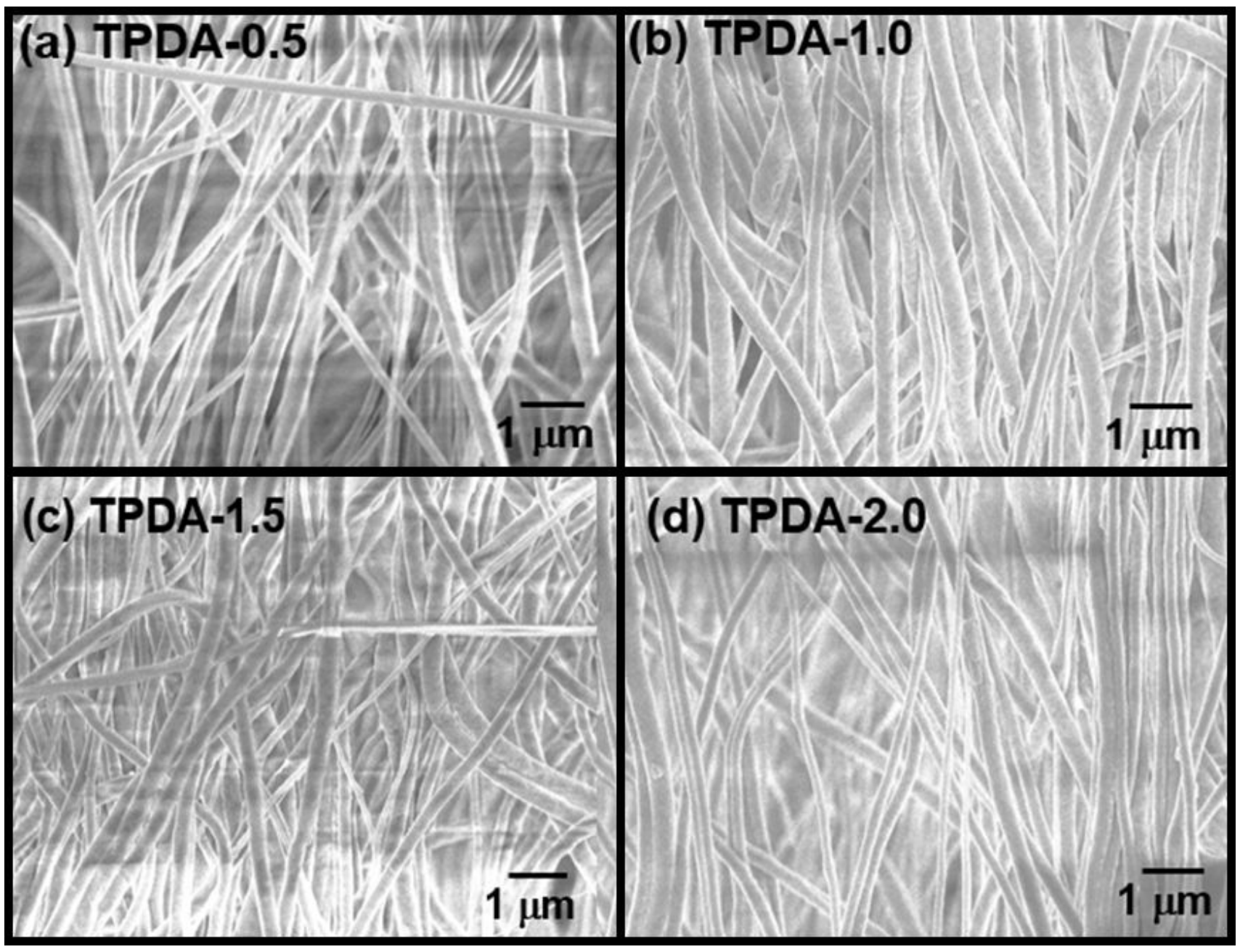

Figure S9. SEM images of the TPDA-based PVDF-HFP nanofibrous membranes.

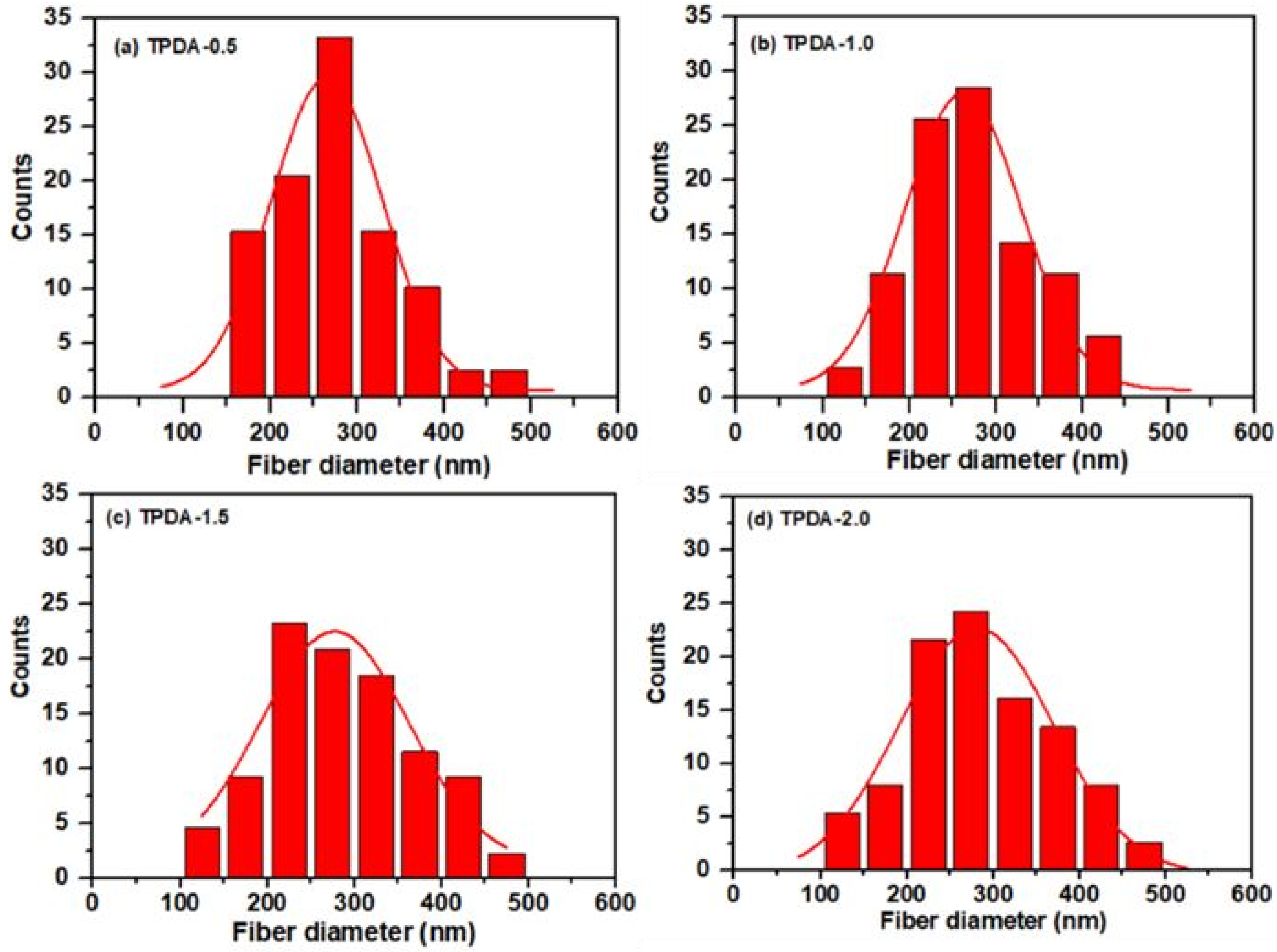

Figure S10. Fiber diameter distribution of the TPDA-based PVDF-HFP nanofibrous membranes.

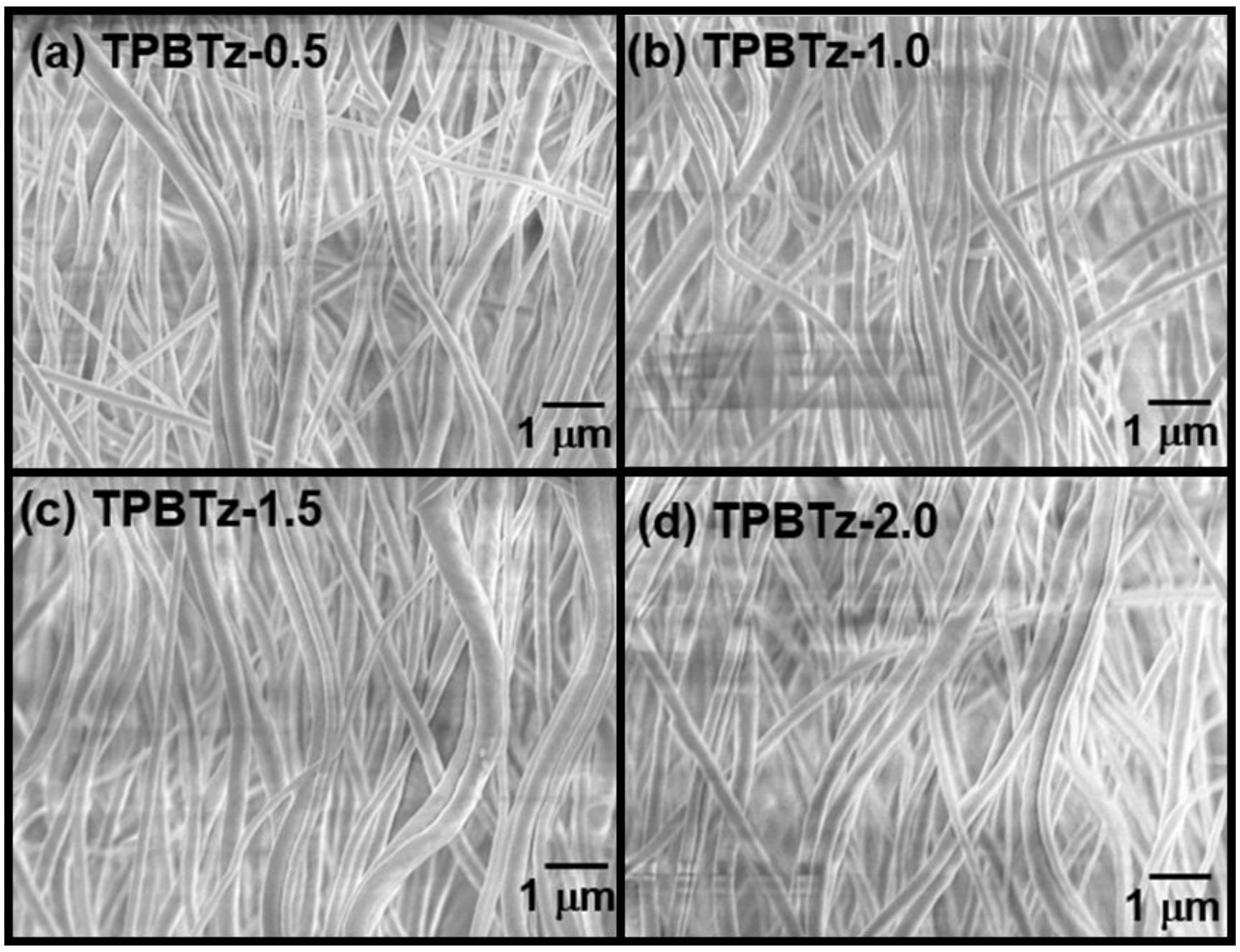

Figure S11. SEM images of the TPBTz-based PVDF-HFP nanofibrous membranes.

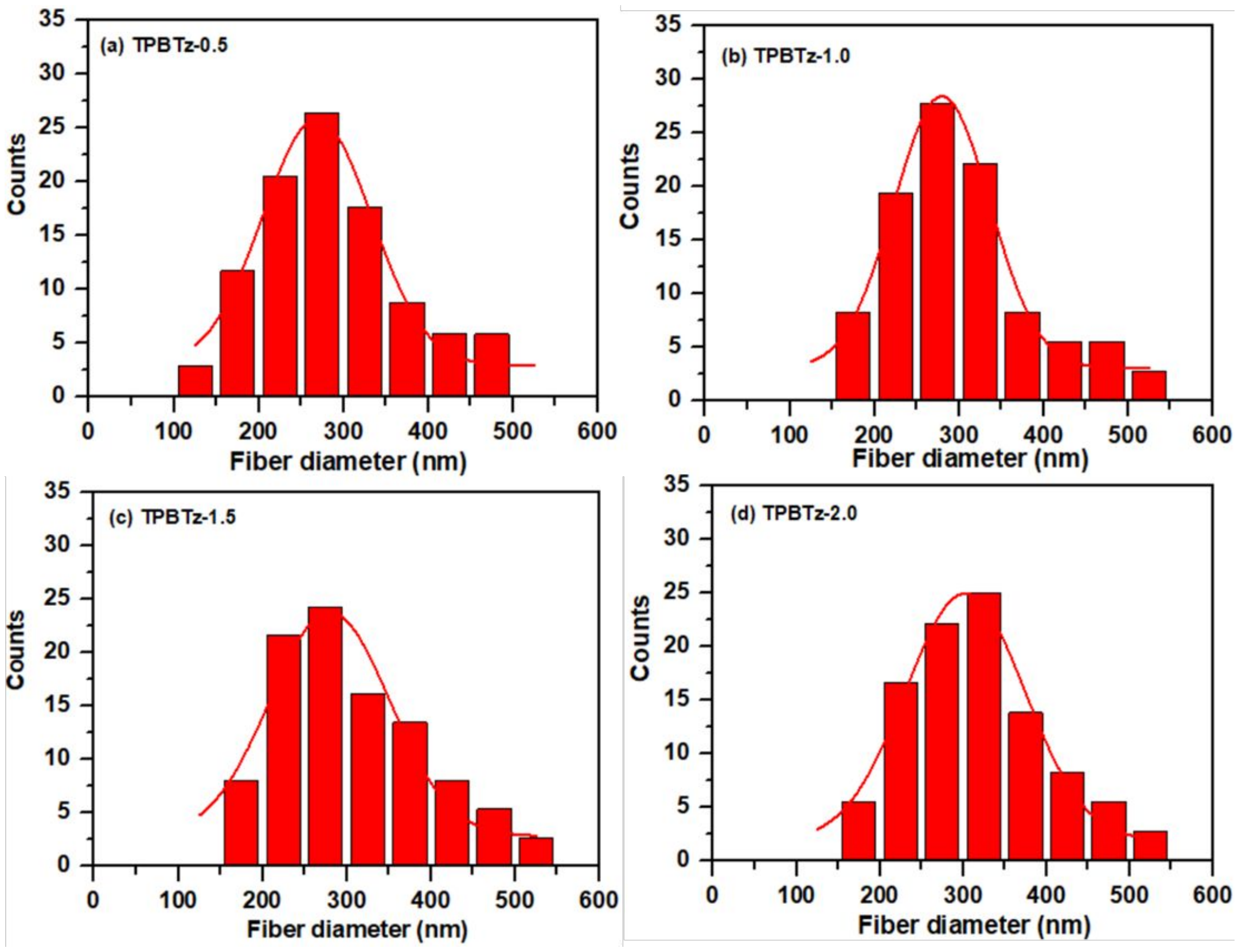

Figure S12. Fiber diameter distribution of the TPBTz-based PVDF-HFP nanofibrous membranes.

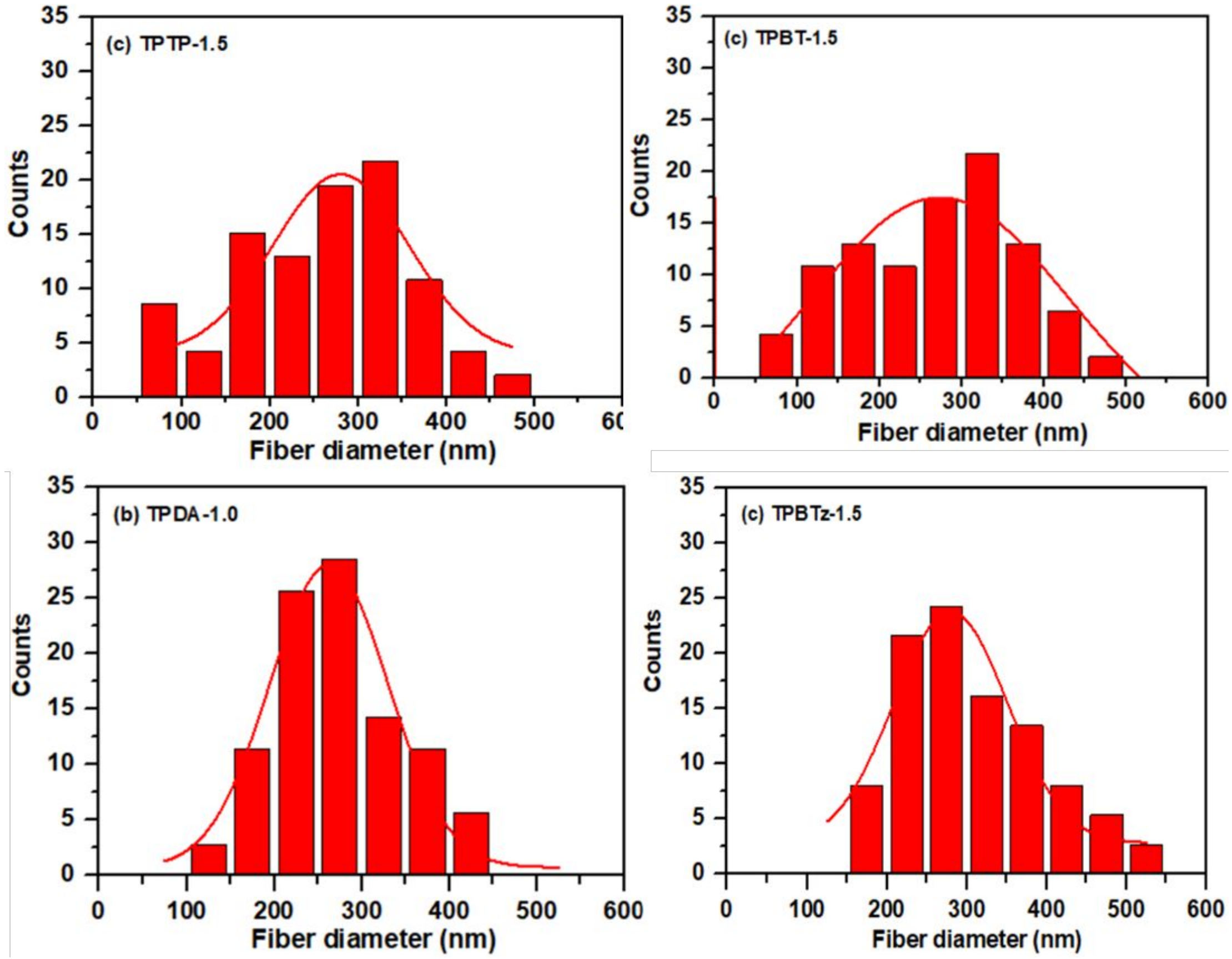

Figure S13. Fiber diameter distribution of the (a) TPTP-1.5, (b) TPBT-1.5, (c) TPDA-1.0, and (d) TPBTz-1.5 nanofibrous membranes.

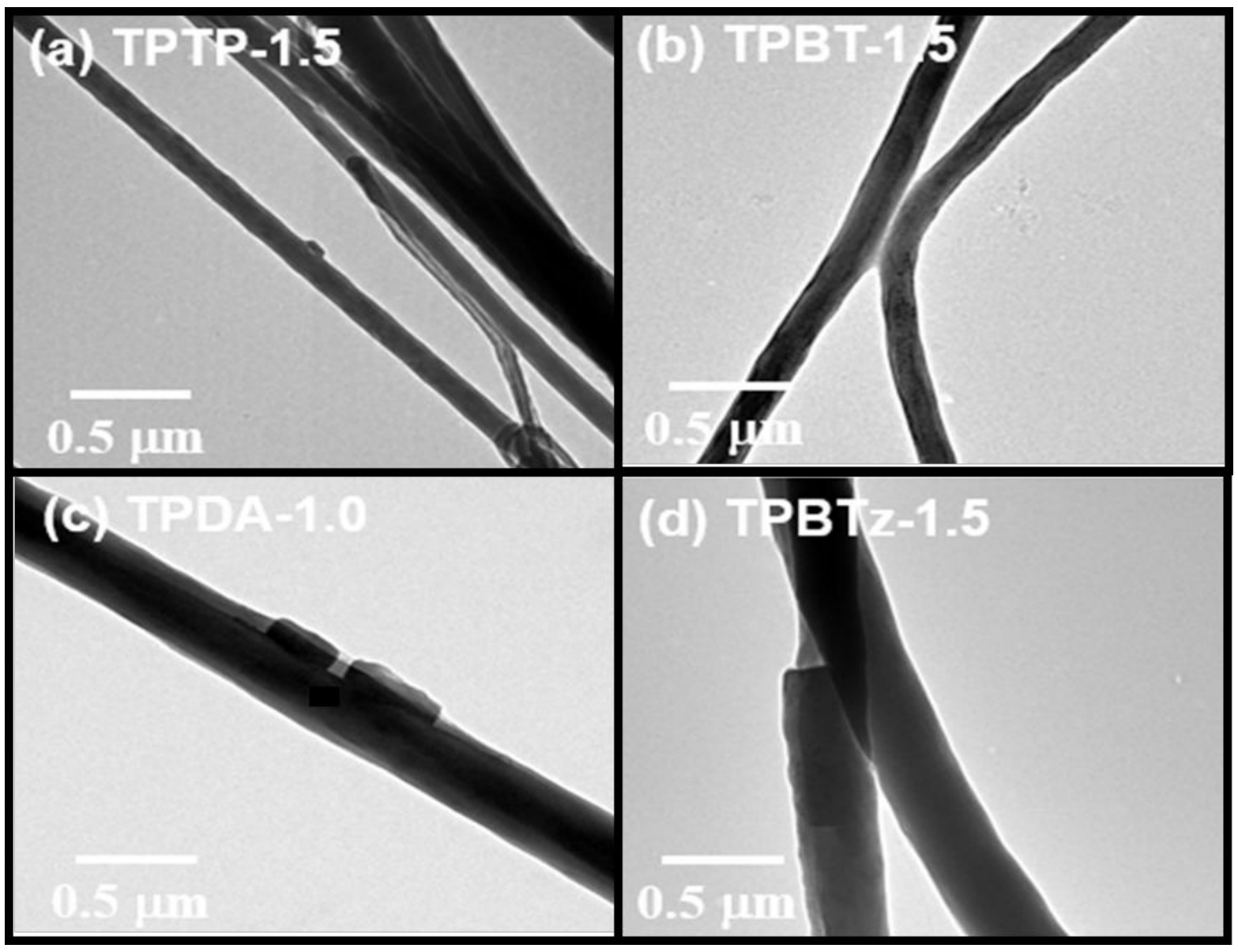

Figure S14. TEM images of the (a) TPTP-1.5, (b) TPBT-1.5, (c) TPDA-1.0, and (d) TPBTz-1.5 nanofibrous membranes.

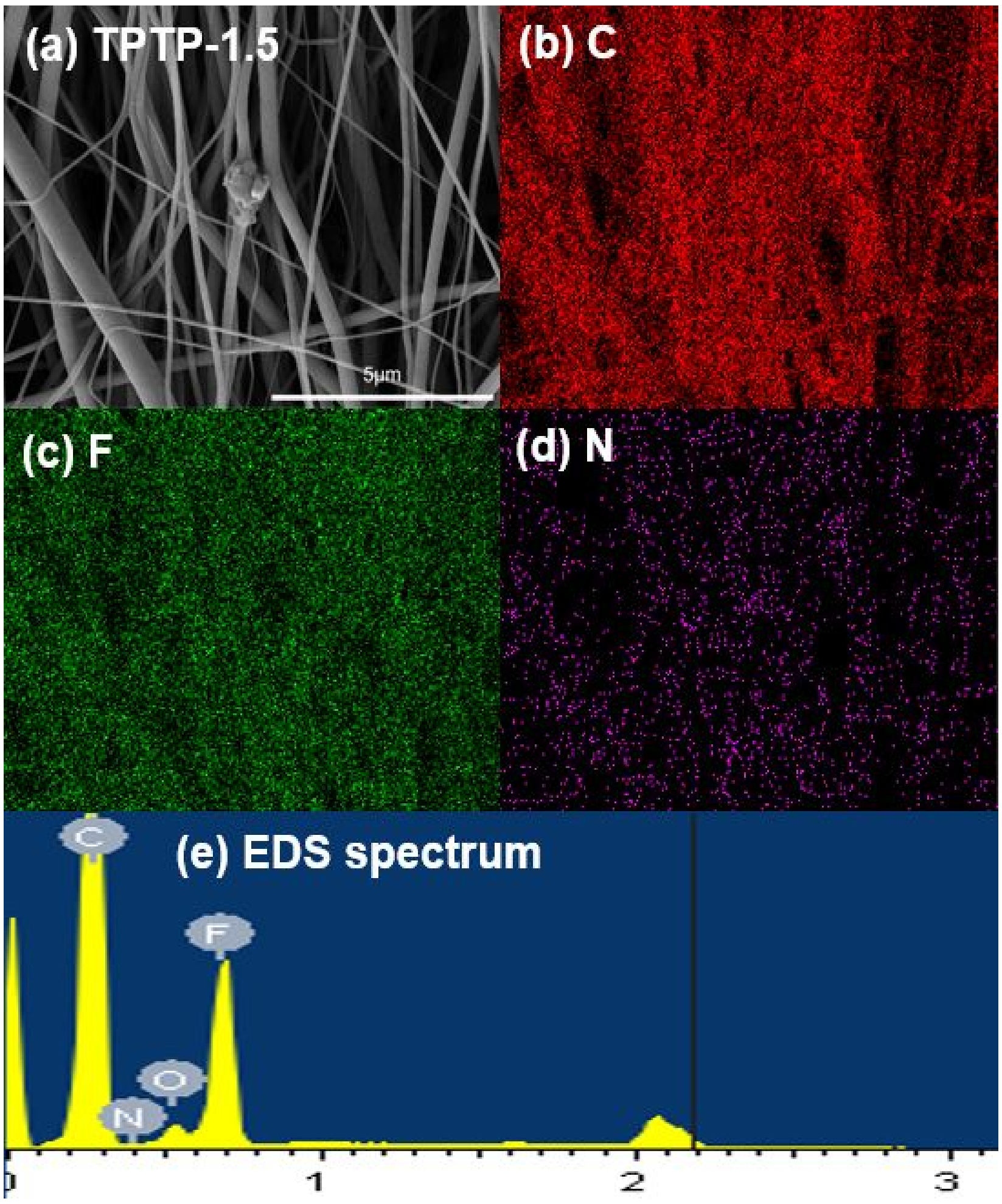

Figure S15. (a) SEM image, (b−d) EDX images of (b) C, (c) F, and (d) N, and (e) EDS spectrum in the TPTP-1.5 nanofibrous membranes.

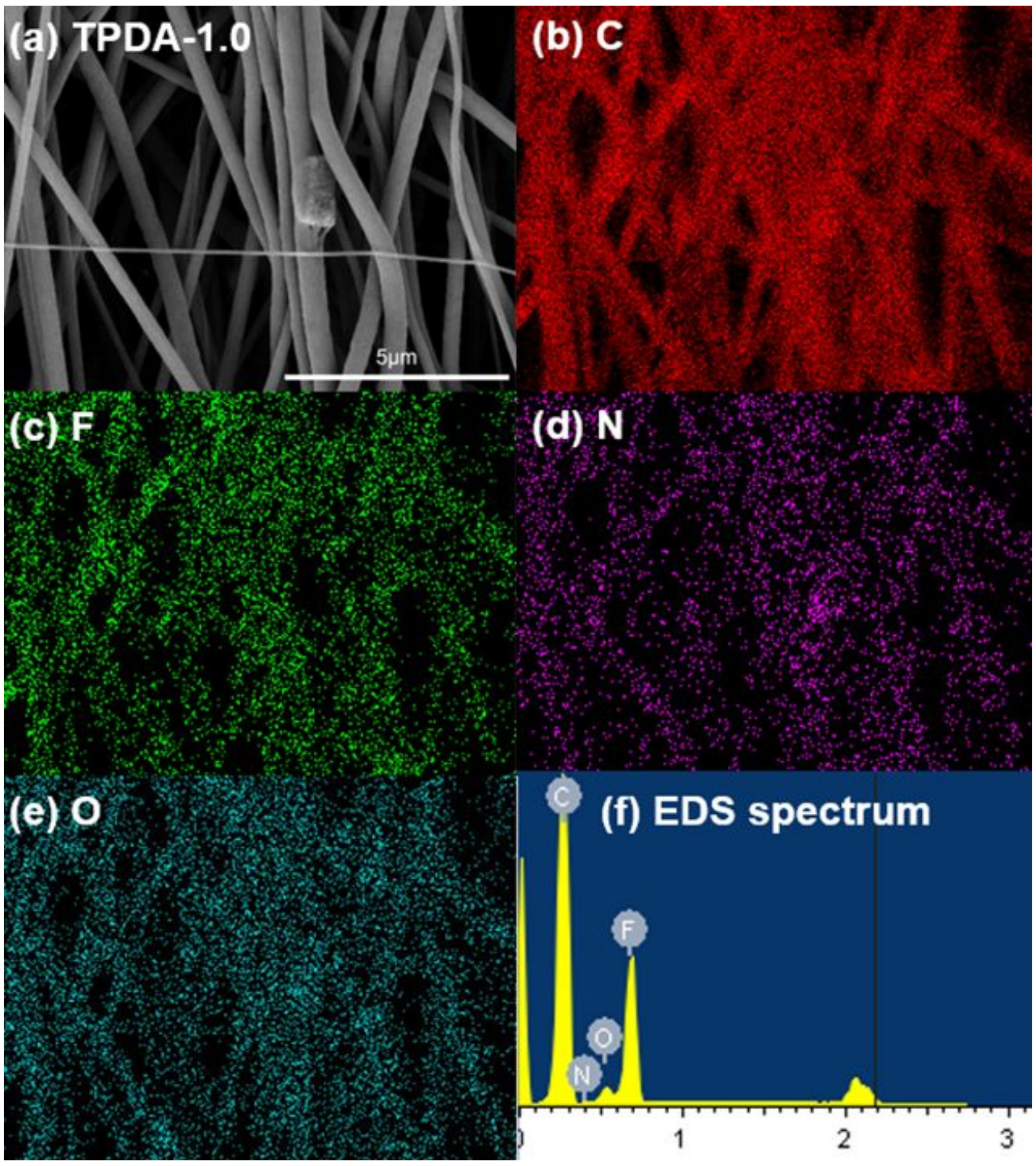

Figure S16. (a) SEM image, (b–d) EDX images of (b) C, (c) F, (d) N, and (e) O, and (f) EDS spectrum in the TPDA-1.0 nanofibrous membranes.

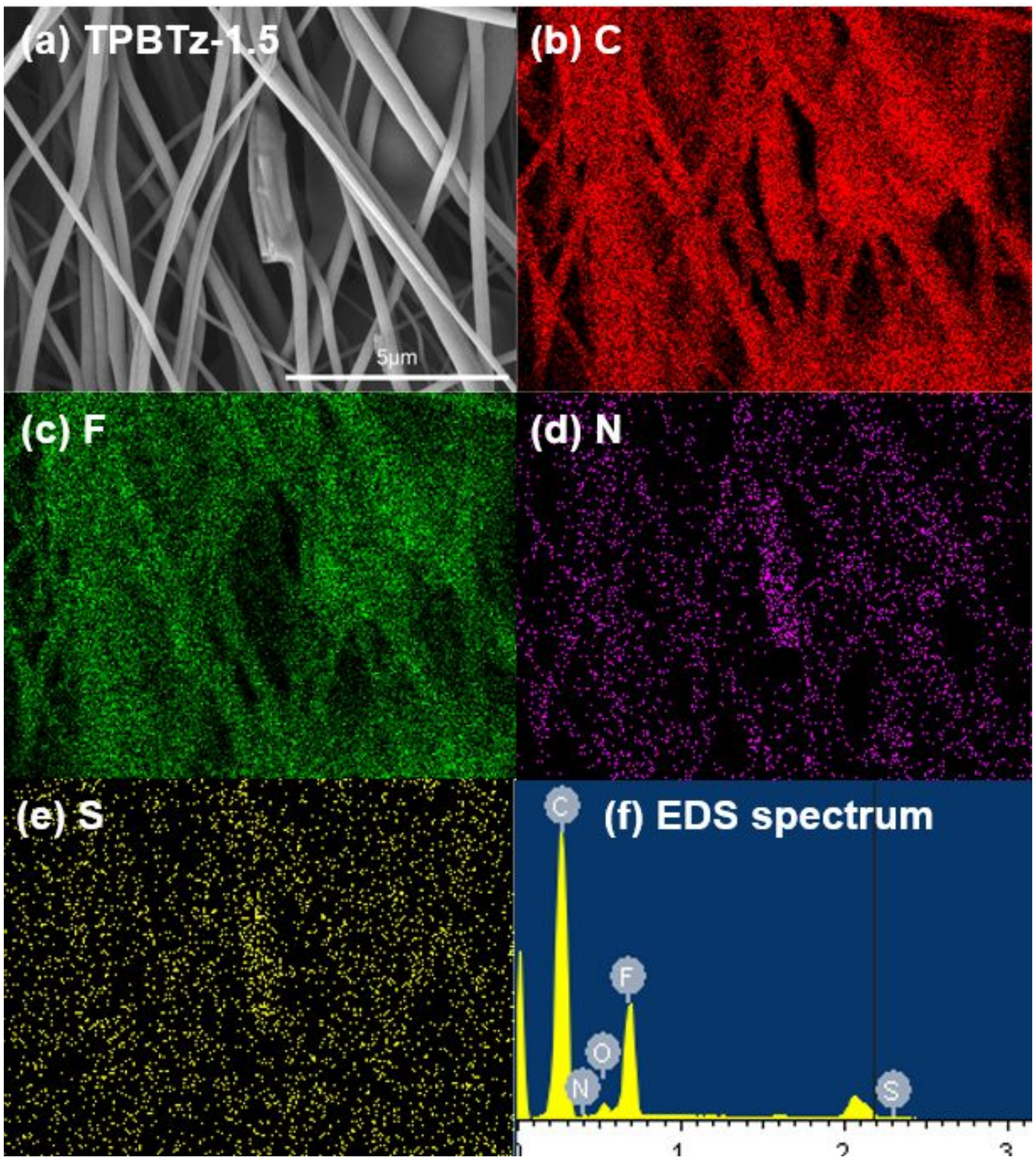

Figure S17. (a) SEM image, (b‒e) EDX images of (b) C, (c) F, (d) N, and (e) S, and (f) EDS spectrum in the TPBTz-1.5 nanofibrous membranes.

Table S2. The β-phase fraction F(β) in the COF/PVDF-HFP nanofibrous membranes.

Sample	COF content in PVDF-HFP (wt. %)	F(β) (%)
Pristine PVDF-HFP	0	81.3
TPTP-0.5	0.5	77.0
TPTP-1.0	1.0	77.1
TPTP-1.5	1.5	75.3
TPTP-2.0	2.0	72.7
TPBT-0.5	0.5	80.1
TPBT-1.0	1.0	79.9
TPBT-1.5	1.5	78.4
TPBT-2.0	2.0	77.9
TPDA-0.5	0.5	82.3
TPDA-1.0	1.0	80.3
TPDA-1.5	1.5	78.1
TPDA-2.0	2.0	78.9
TPBTz-0.5	0.5	81.6
TPBTz-1.0	1.0	80.8
TPBTz-1.5	1.5	79.0
TPBTz-2.0	2.0	75.3

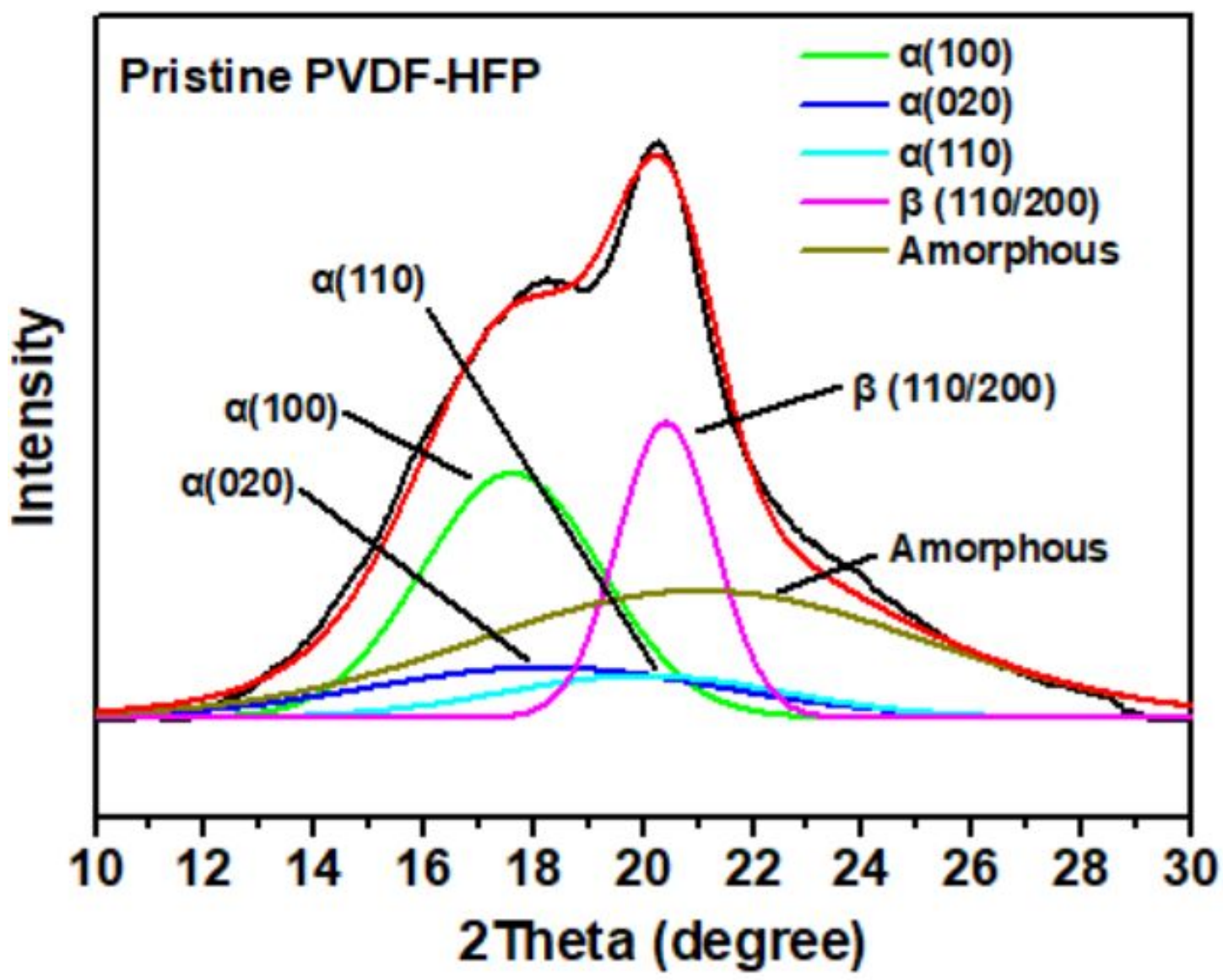

Figure S18. XRD image of the pristine PVDF-HFP nanofibrous film.

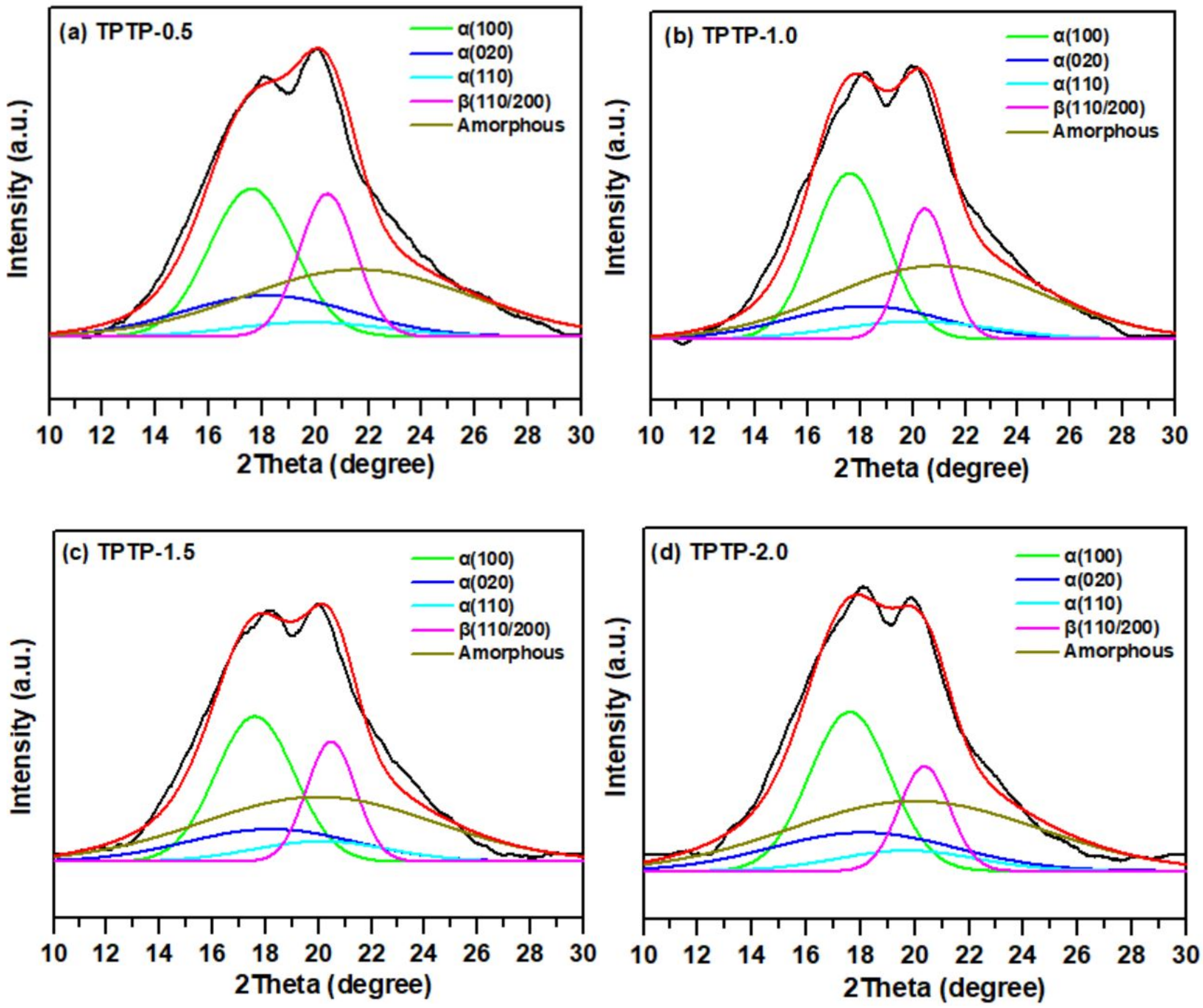

Figure S19. XRD image of the TPTP-COF/PVDF-HFP nanofibrous film.

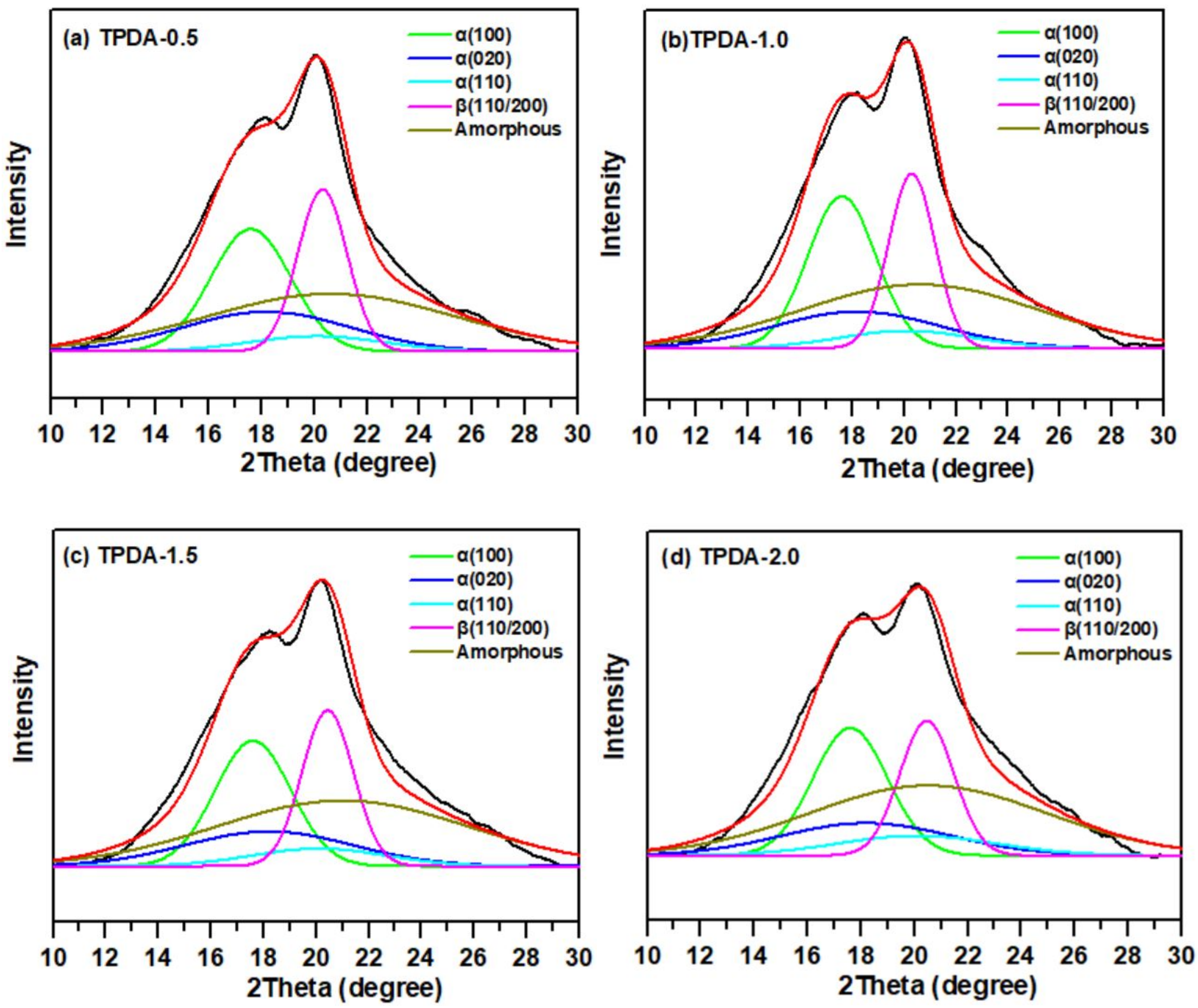

Figure S20. XRD image of the TPDA-COF/PVDF-HFP nanofibrous film.

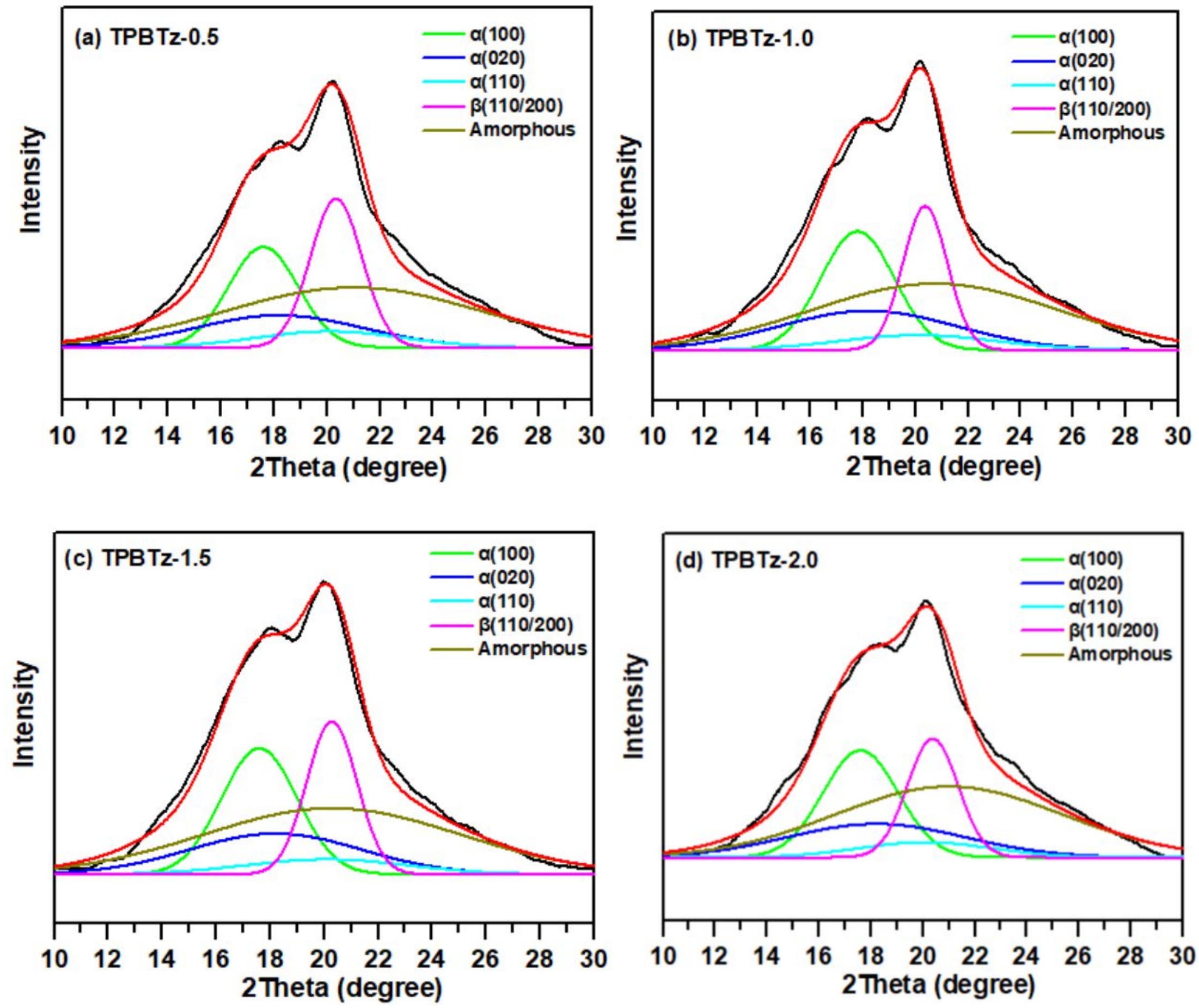

Figure S21. XRD image of the TPBTz-COF/PVDF-HFP nanofibrous film.

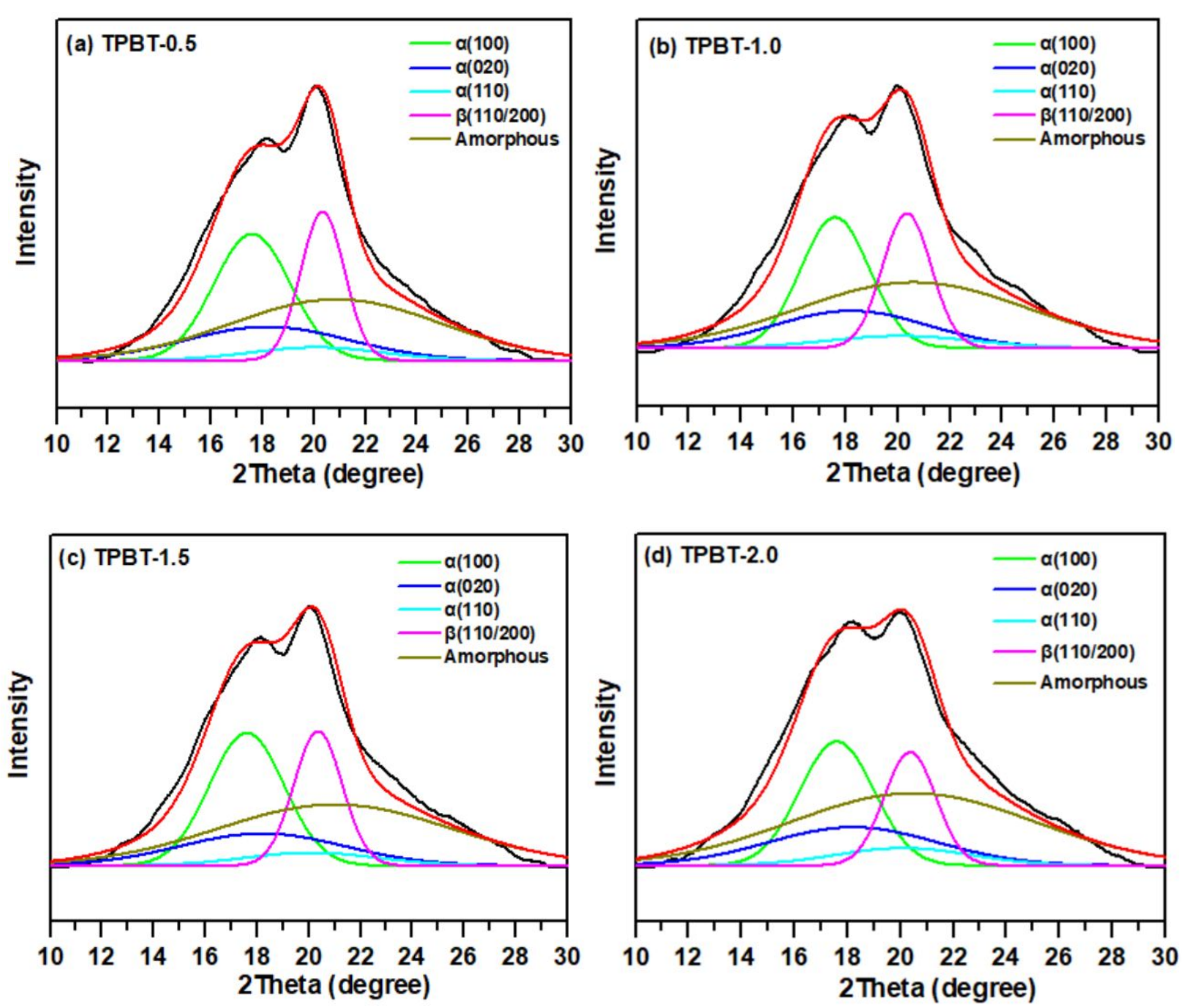

Figure S22. XRD images of the TPBT-COF/PVDF-HFP nanofibrous films.

Table S3. Phase contents (α-phase, β-phase, and amorphous phase) in the COF/PVDF-HFP nanofibrous membranes.

Sample	α (%)	β (%)	Amorphous (%)
Pristine PVDF-HFP	46.1	18.0	35.9
TPTP-0.5	47.5	16.7	35.8
TPTP-1.0	49.1	15.6	35.3
TPTP-1.5	49.3	15.1	35.6
TPTP-2.0	50.2	14.5	35.3
TPBT-0.5	45.7	17.9	36.4
TPBT-1.0	45.9	17.4	36.7
TPBT-1.5	46.3	17.2	36.5
TPBT-2.0	47.6	15.8	36.6
TPDA-0.5	46.4	18.5	35.1
TPDA-1.0	46.3	18.2	35.5
TPDA-1.5	46.4	17.8	35.8
TPDA-2.0	46.1	17.7	36.2
TPBTz-0.5	46.6	17.8	35.6
TPBTz-1.0	47.1	17.5	35.4
TPBTz-1.5	47.2	17.3	35.5
TPBTz-2.0	47.4	16.8	35.8

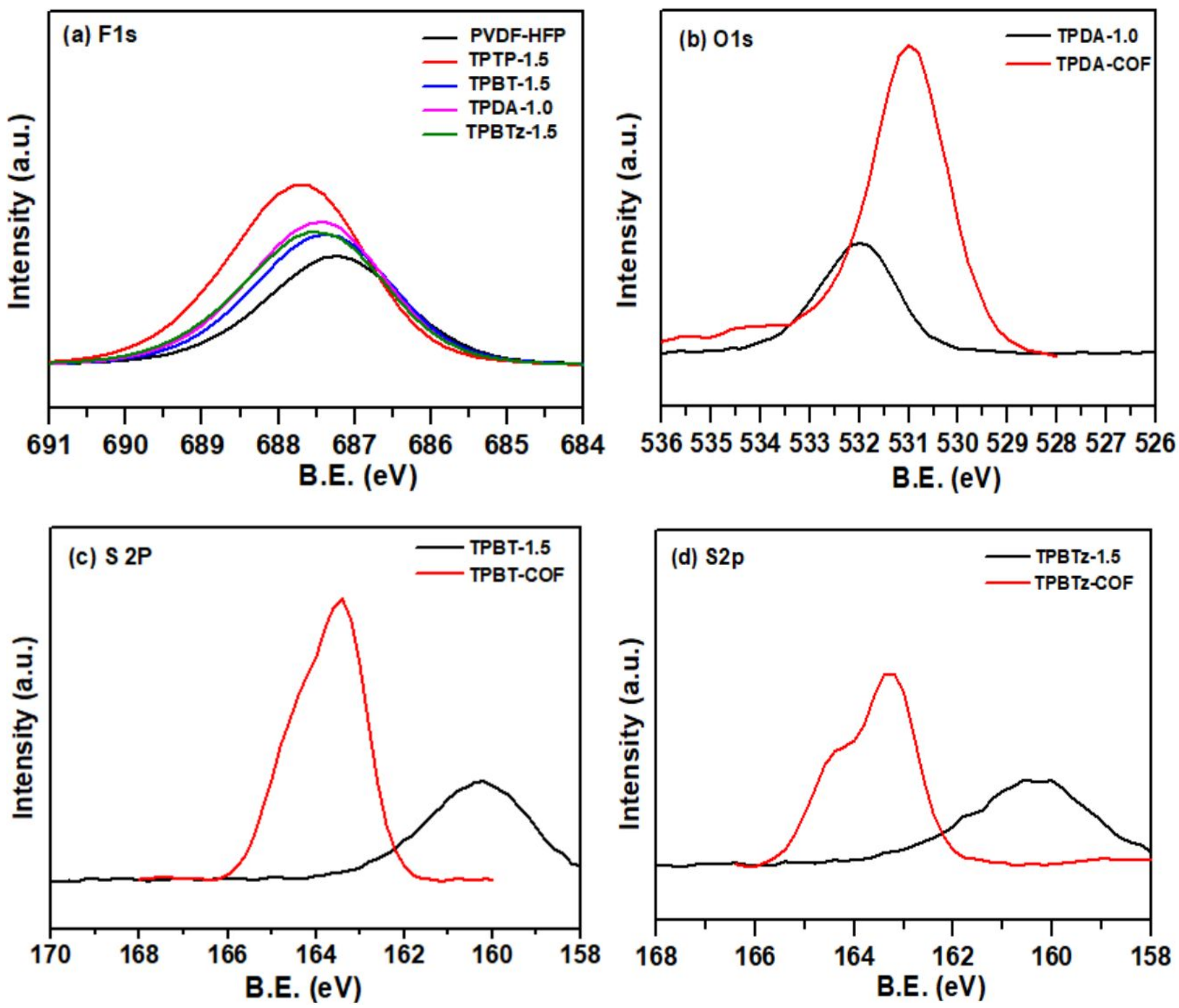

Figure S23. XPS spectra of COF and COF/PVDF-HFP composite films: (a) F 1s spectra of PVDF-HFP, TPTP-1.5, TPBT-1.5, TPDA-1.0, and TPBTz-1.5; (b) O 1s spectra of TPDA-COF and TPDA-1.0; (c) S 2p spectra of TPBT-COF and TPBT-1.5; (d) S 2p spectra of TPBTz-COF and TPBTz-1.5.

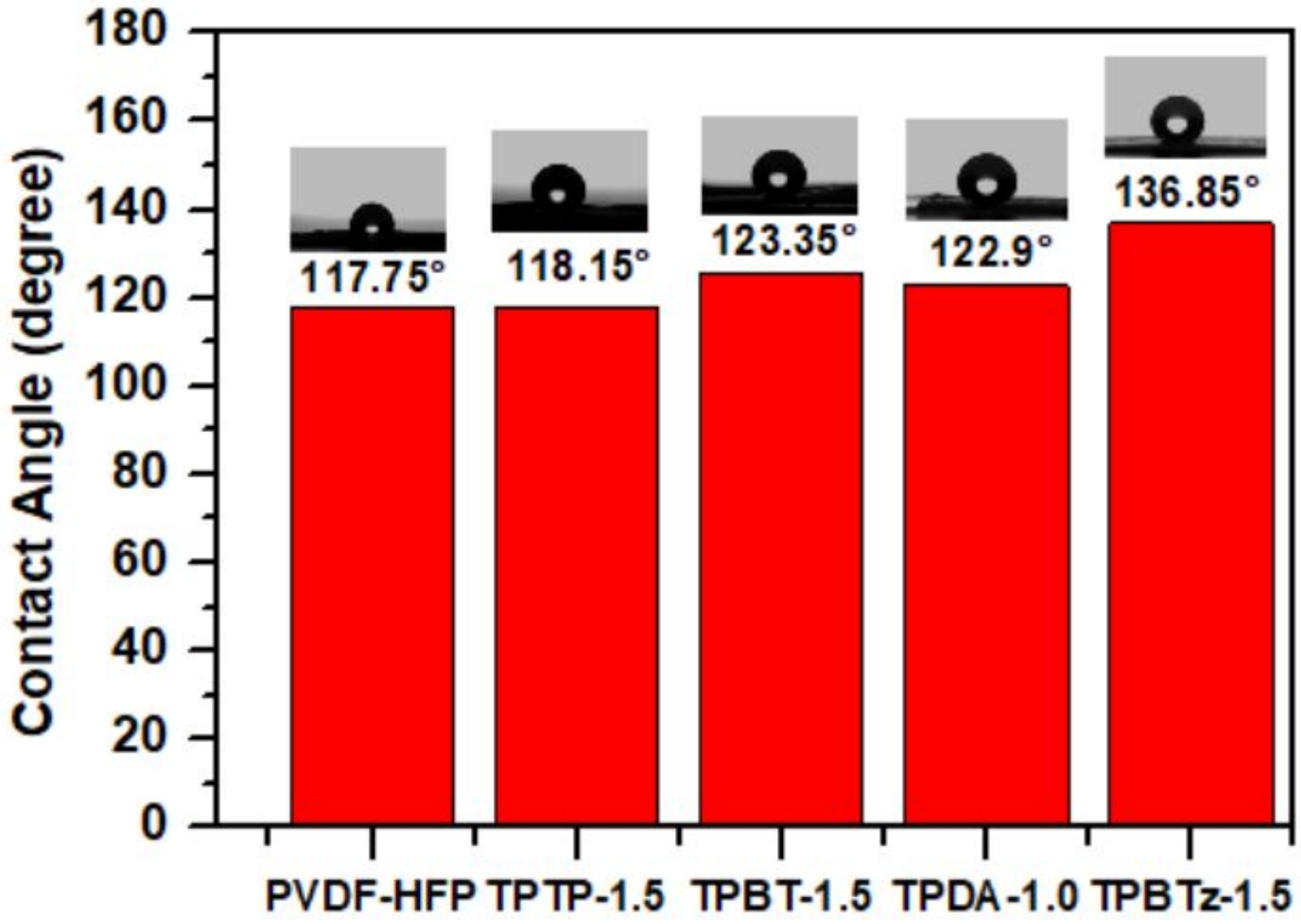

Figure S24. CAs of the COF/PVDF-HFP nanofibrous membranes.

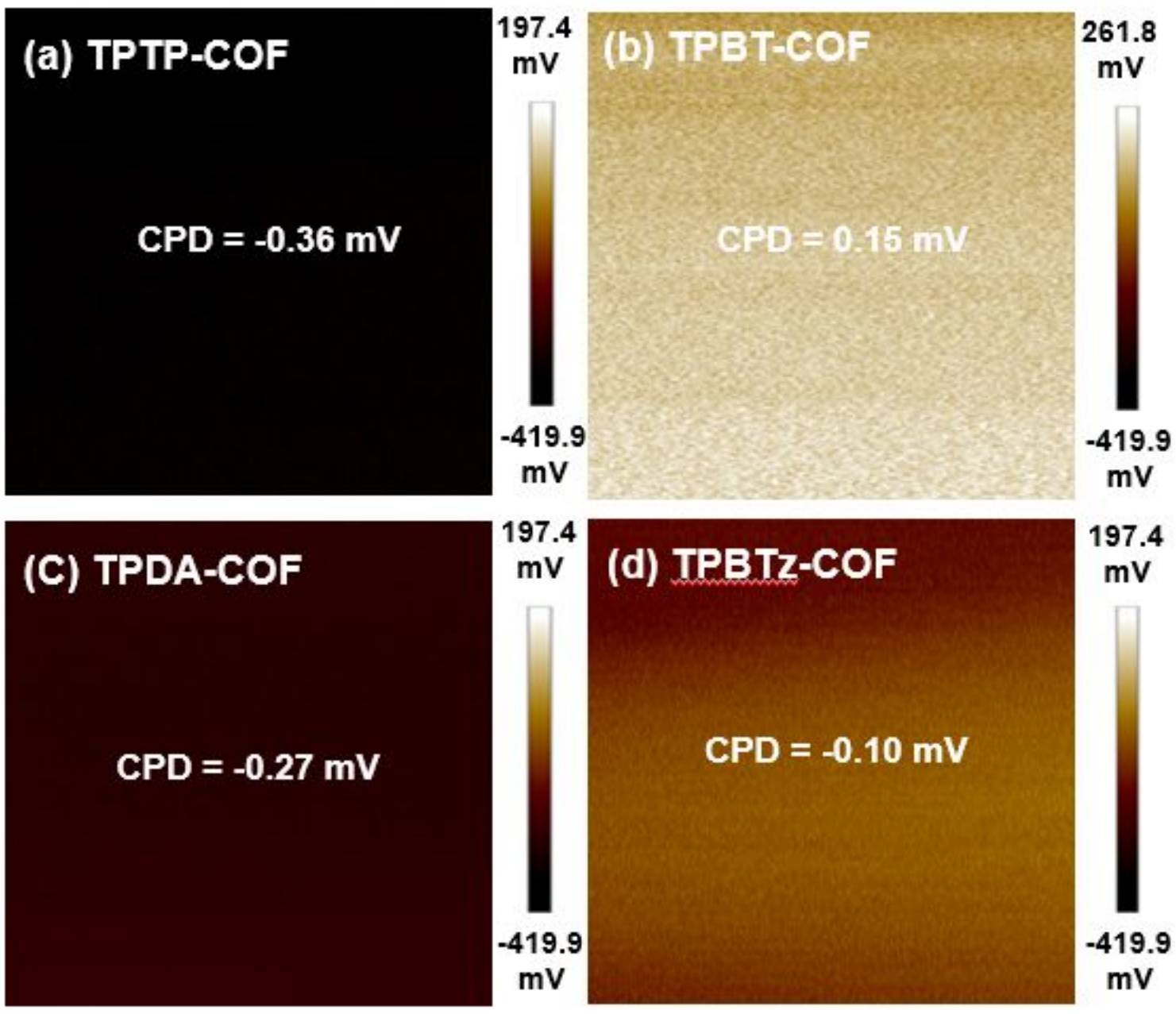

Figure S25. KPFM surface potential maps (100×100 nm²) of the TPTP-COF, TPBT-COF, TPDA-COF, and TPBTz-COF samples.

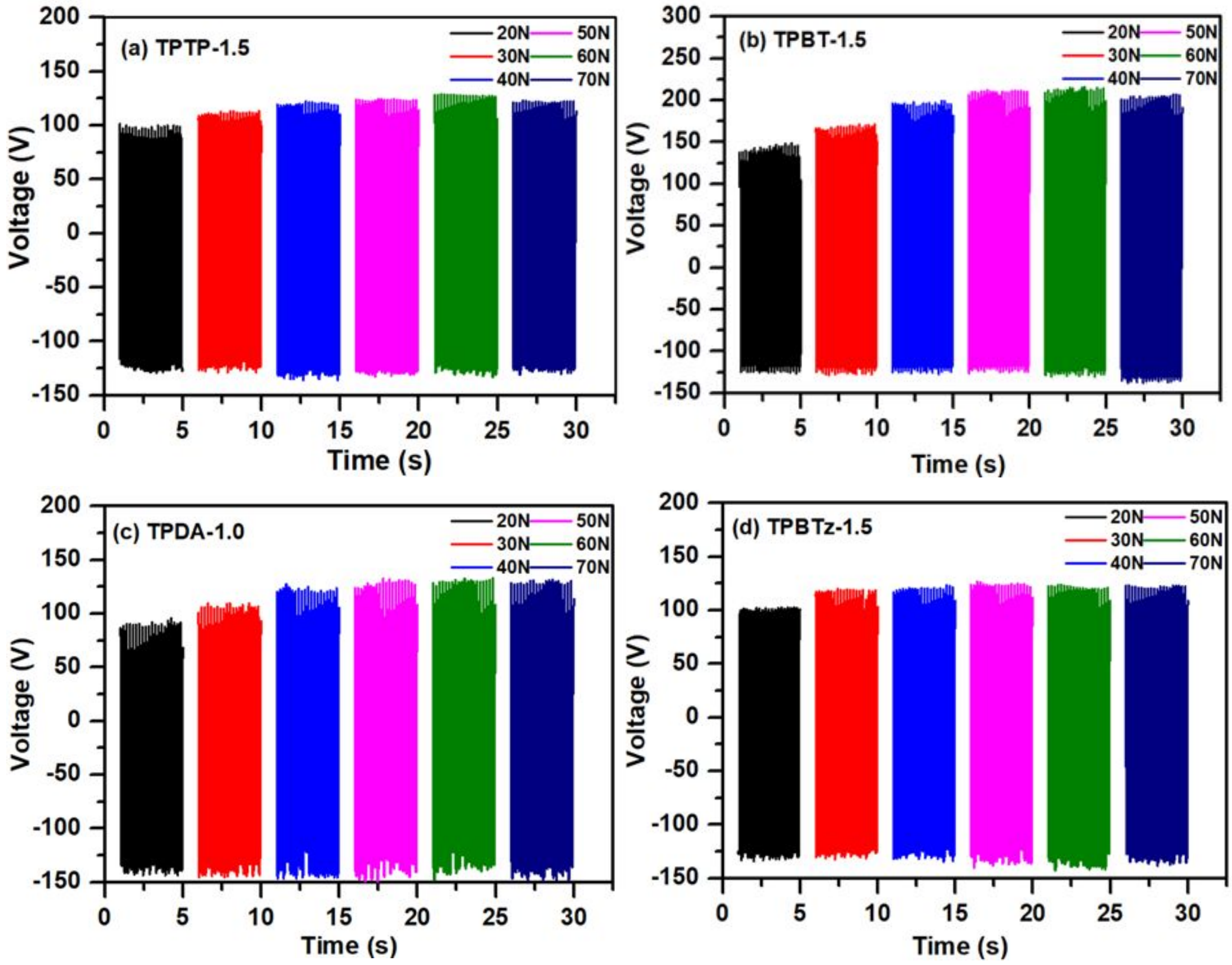

Figure S26. V_{OC} of the COF/PVDF-HFP based TENGs measured at 5 Hz under various applied forces [(a) TPTP-1.5 TENG; (b) TPBT-1.5 TENG; (c) TPDA-1.0 TENG; (d) TPBTz-1.5 TENG].

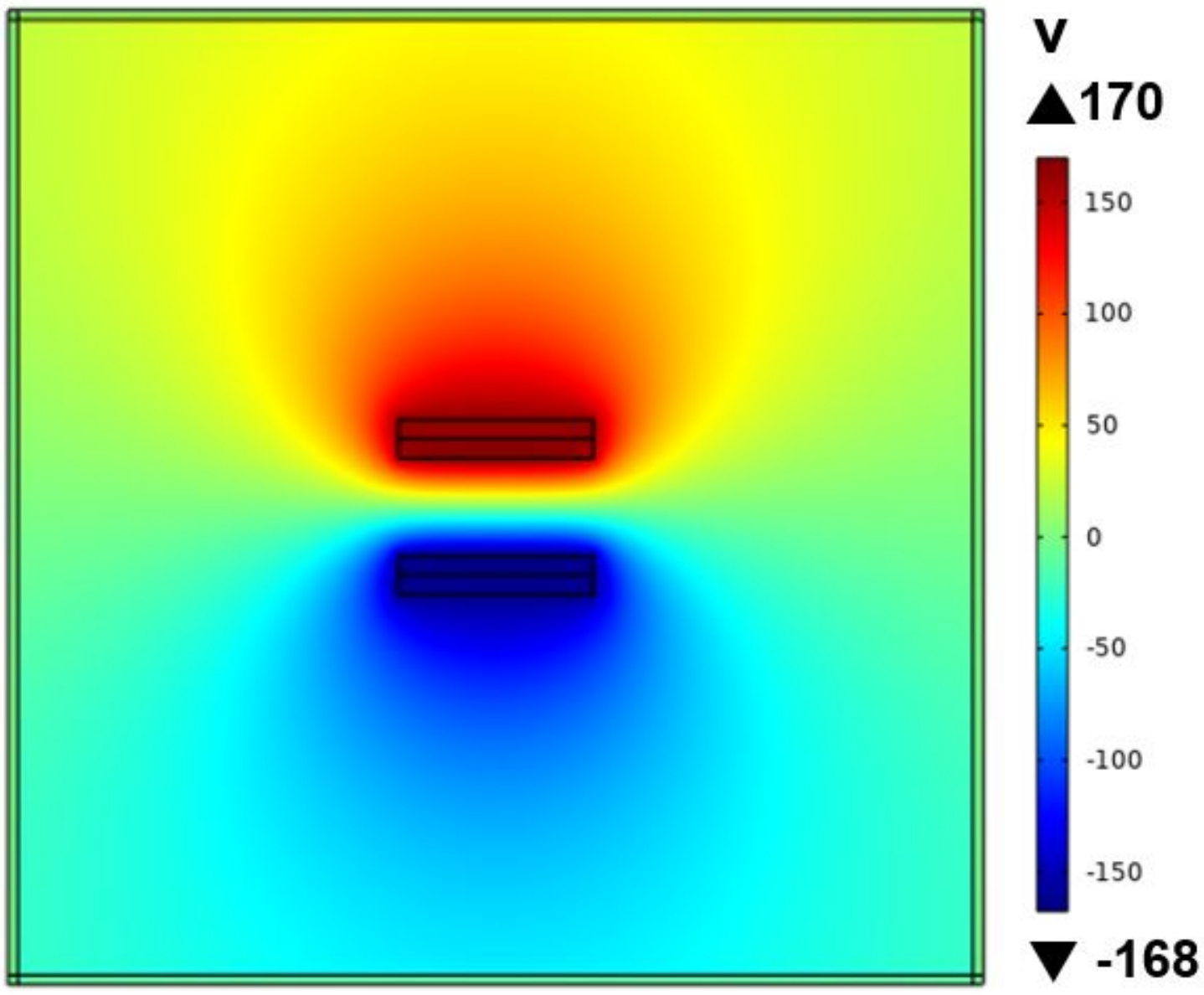

Figure S27. the COMSOL simulation results of the potential distribution in the TPBT-1.5 TENG with 10 mm separation distances.

Table S4. Simulation parameters for the potential distribution during TENG operation.

Global definition：

Parameter name	symbol	value	unit	position	size
**Rectangular** 1	r 1	[100,100,1]		{50,50}	W：100<br>H：100
Down electrode	r 2	[20,2,1]		{90,90}	W：20<br>H：2
Down triboelectric layer	r 3	[20,2,1]	mm	{90,92}	W：20<br>H：2
Top triboelectric layer	r 4	[20,2,1]		{90,94+y1}	W：20<br>H：2
Top electrode	r 5	[20,2,1]		{90,96+y1}	W：20<br>H：2

Global definition：

name	expression	value	description
y1	5e-7[m]	5E−7 m	Displacement parameter

Materials parameter：

Material	symbol	value	unit	description
Air [gas]	ϵ_r	1		relative permittivity
Copper [solid,181 nm thin film]	ϵ_r	1E8		relative permittivity
TPBT-1.5	ϵ_r	12.5	-	relative permittivity
TPU	ϵ_r	4.5		relative permittivity

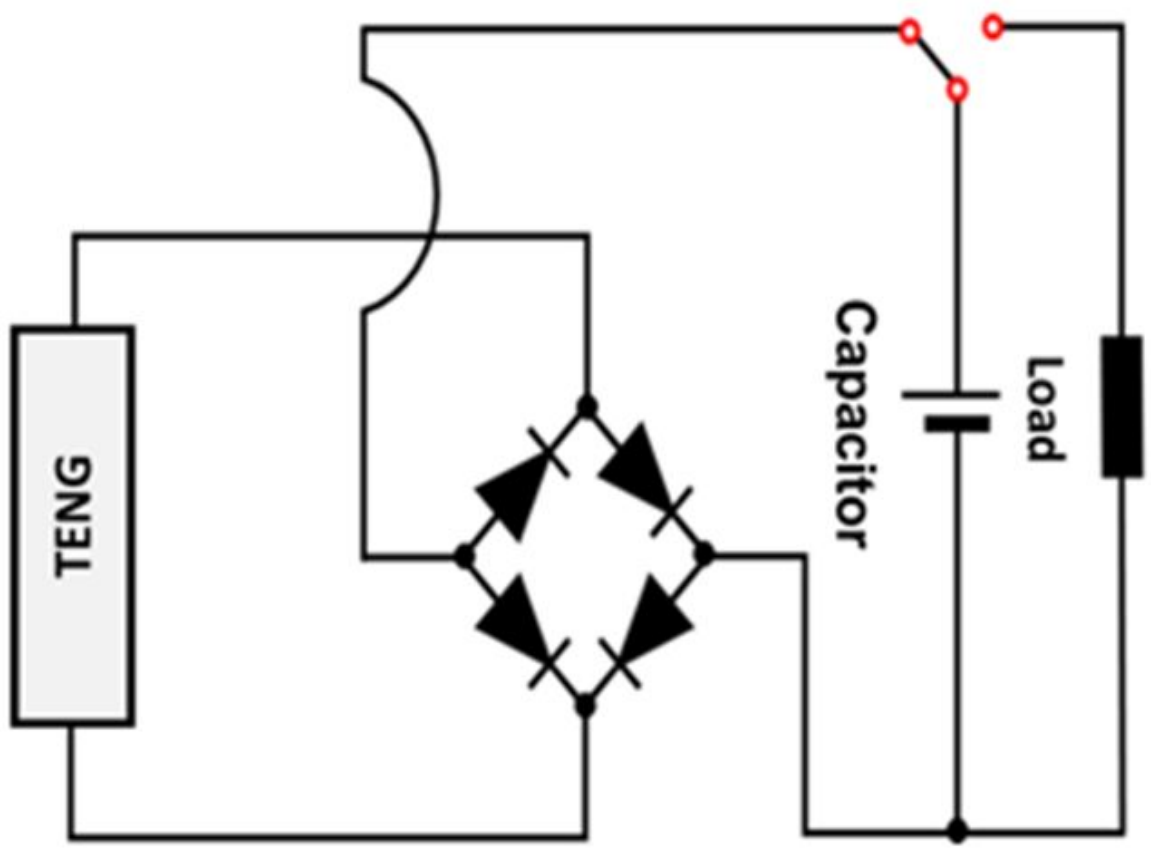

Figure S28. Equivalent circuit diagram for charging commercial capacitors with different capacitances using the TENG.

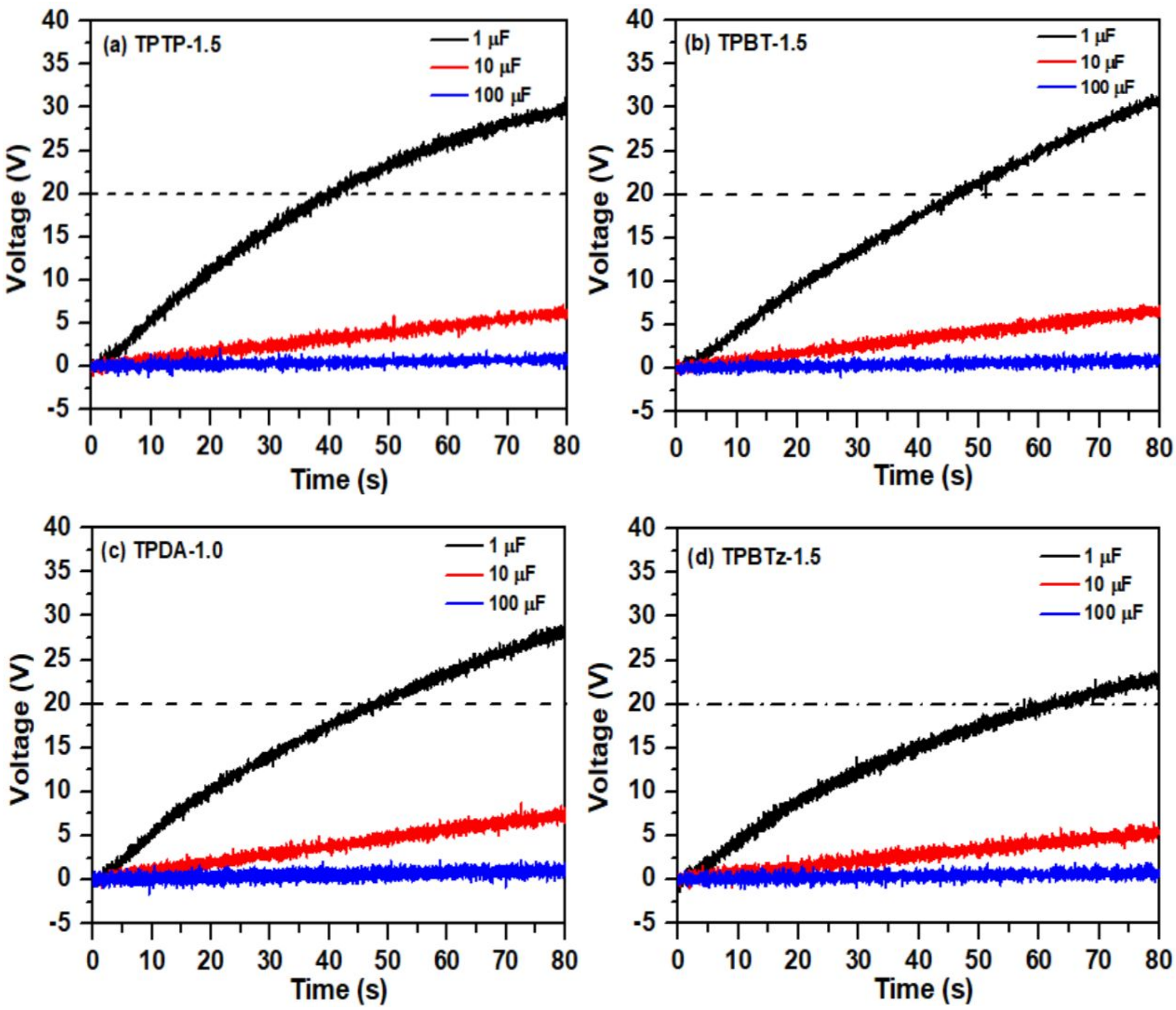

Figure S29. Charging characteristics of commercial capacitors with different capacitances using COF/PVDF-HFP-based TENGs: voltage evolution as a function of

charging time.

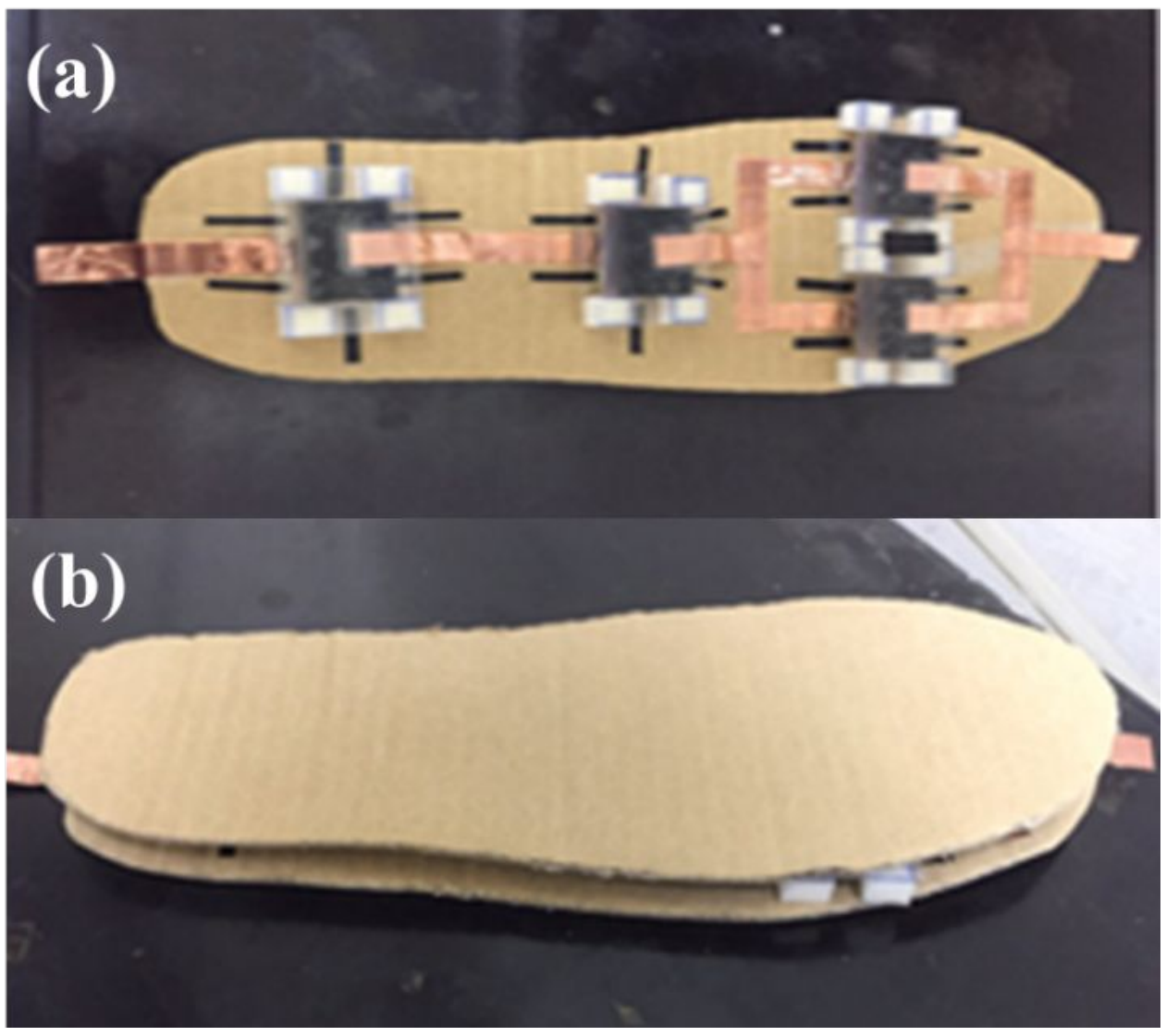

Figure S30. Power-generating insole assembled by connecting four TPBT-1.5 TENGs in series.
